# LncRNA EUDAL shapes tumor cell response to hypoxia-induced constitutive EGFR activation and promotes chemoresistance in oral cancer

**DOI:** 10.1038/s41368-025-00396-2

**Published:** 2025-09-12

**Authors:** Shengkai Chen, Zhenlin Dai, Jianbo Shi, Mengyu Rui, Zhiyuan Zhang, Qin Xu

**Affiliations:** 1 Department of Oral and Maxillofacial-Head and Neck Oncology, Ninth People’s Hospital, Shanghai Jiao Tong University, School of Medicine, Shanghai, China; 2 Shanghai Key Laboratory of Stomatology & Shanghai Research Institute of Stomatology, Shanghai, China; 3 National Clinical Research Center for Oral Disease, Shanghai, China; 4 Department of Oral and Maxillofacial Surgery, Zhongshan Hospital, Fudan University, Shanghai, China

**Keywords:** Oral cancer, Cancer microenvironment, Tumour biomarkers

## Abstract

Hypoxia and aberrant activation of epidermal growth factor receptor (EGFR) are considered important features of various malignancies. However, whether hypoxia can directly trigger EGFR activation and its clinical implications remain unclear. In this study, we demonstrated that in oral cancer, a typical hypoxic tumor, hypoxia can induce chronic but constitutive phosphorylation of wild-type EGFR in the absence of ligands. Oral cancer cell lines exhibit different EGFR phosphorylation responses to hypoxia. In hypoxic HN4 and HN6 cells, ubiquitination-mediated endocytosis, lysosomal sorting, and degradation lead to low levels of EGFR phosphorylation. However, in CAL-27 and HN30 cells, a novel HIF-1α-induced long noncoding RNA (lncRNA), EUDAL, can compete with the E3 ligase/adaptor complex c-Cbl/Grb2 for binding to EGFR, stabilizing phosphorylated EGFR (pEGFR) and resulting in sustained activation of EGFR and its downstream STAT3/BNIP3 signaling. STAT3/BNIP3-mediated autophagy leads to antitumor drug resistance. A high EUDAL/EGFR/STAT3/autophagy pathway activation predicts poor response to chemotherapy in oral cancer patients. Collectively, hypoxia can induce noncanonical ligand-independent EGFR phosphorylation. High EUDAL expression facilitates sustained EGFR phosphorylation in hypoxic tumor cells and leads to autophagy-related drug resistance.

## Introduction

Aberrant activation of the epidermal growth factor receptor (EGFR) is considered a feature of various malignancies. Sustained EGFR activation activates downstream signaling networks and contributes to tumor cell growth, survival, and motility^1^. The binding of epidermal growth factor (EGF) ligand family members to induce EGFR phosphorylation and activation, referred to as ‘canonical’ EGFR activation, has been best characterized^2^. Ligand binding can facilitate the dimerization of the receptor and promote the phosphorylation of tyrosine residues in its kinase domain and C-terminus, resulting in the activation of EGFR and its downstream signaling pathways^3^.

Recent evidence indicates that EGFR activation may occur even in the absence of ligands, known as ‘noncanonical’ EGFR activation. Spontaneous EGFR activation in the absence of ligands can be induced by exposure to radiation or ultraviolet rays or by specific mutations^1,4–6^. In tumors, canonical and noncanonical EGFR activation may trigger mutually exclusive downstream signaling and lead to distinct therapeutic responses. Ligand-mediated activation of EGFR may confer either chemosensitivity or chemoresistance in different types of tumors^7,8^. Noncanonical EGFR activation usually induces chemotherapy resistance^9,10^, but it may lead to sensitivity to EGFR-targeted therapies^11–13^. Unlike those of ligand-stimulated EGFR activation, the molecular mechanisms, cellular responses, and biological effects of noncanonical EGFR activation in tumors are incompletely elucidated.

Hypoxia is a common characteristic of human solid tumors and is closely associated with resistance to multiple therapeutic modalities^14,15^. In general, head and neck, cervical, and lung tumors are the most hypoxic^16^. Tumor hypoxia has been recognized not only as an outcome of uncontrolled cellular growth in the tumor microenvironment but also as an active participant in tumor progression. Although recent studies have shown that hypoxia can induce the expression of EGFR^17^, the role of hypoxia in directly triggering EGFR activation remains incompletely characterized. Given that the EGFR activation status is more important than the total EGFR expression level during tumor progression^18^, it is necessary to define the exact role of hypoxia in ligand-independent EGFR activation.

Long noncoding RNAs (lncRNAs) are RNAs that lack protein-coding ability and contain more than 200 nucleotides; these RNAs are associated with various malignant behaviors^19^. LncRNAs play critical roles in regulating cellular functions via interactions with DNA, RNA, or protein partners^20^. However, compared with those of protein-coding RNAs, the precise mechanisms by which lncRNAs perform their biological functions in tumor cells remain unclear.

In this study, we demonstrated that in oral cancer cells harboring wild-type (wt) EGFR, hypoxia can induce ligand-independent EGFR phosphorylation. A previously uncharacterized lncRNA, EUDAL, can protect EGFR from ubiquitination by competing with the c-Cbl/Grb2 complex for binding to EGFR, thereby affecting the endocytosis and lysosomal degradation of hypoxia-induced phosphorylated EGFR (pEGFR). High expression of EUDAL facilitates sustained phosphorylation of EGFR in hypoxic tumor cells and subsequently activates downstream STAT3/BNIP3 signaling, which leads to autophagy-related drug resistance.

## Results

### Hypoxia promotes EGFR dimerization and induces ligand-independent constitutive EGFR phosphorylation

**Fig. 1:**
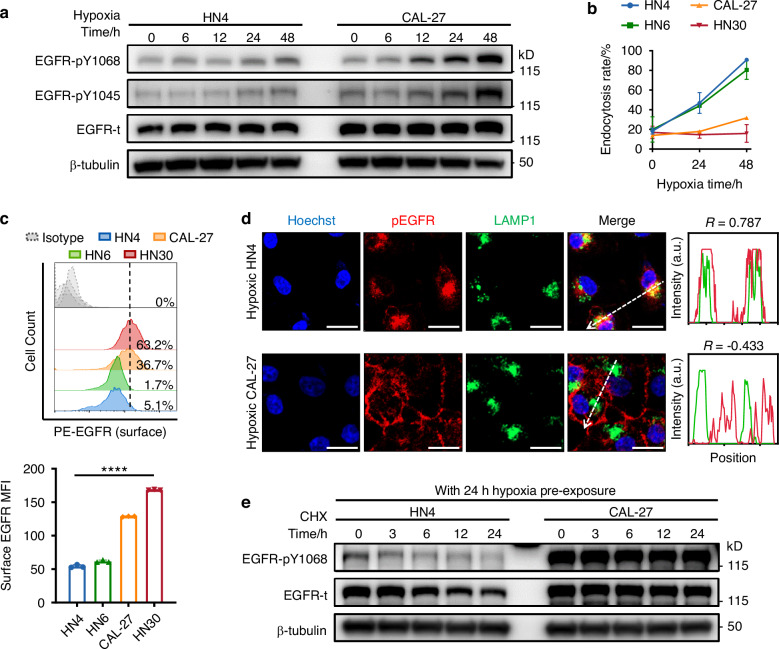
Hypoxia induces ligand-independent constitutive EGFR phosphorylation via inhibition of its endocytosis and lysosomal degradation. **a** Hypoxia-induced chronic but constitutive EGFR phosphorylation on a series of tyrosine residues. **b** pEGFR endocytosis rate in hypoxic HN4, CAL-27, HN6, and HN30 cells (*n* = 3). **c** Flow cytometric analysis of membrane-localized EGFR on hypoxic HN4, CAL-27, HN6, and HN30 cells (*n* = 3). Percentage of cells with high EGFR intensity was indicated. **d** The colocalization of pEGFR with the lysosome marker LAMP1 in hypoxic HN4 and CAL-27 cells, as shown by confocal microscopy images. The colocalization of pEGFR (red) and LAMP1 (green) was estimated with plot profile using ImageJ. Correlation coefficient *R* > 0.6 was considered well-collocated, while *R* < 0.6 was taken as poorly collocated. Bar, 20 µm. **e** Representative immunoblot images and half-life analysis of pEGFR and total EGFR levels of HN4 and CAL-27 cells after CHX treatment for the indicated time periods. Data were from representative results of at least three independent experiments. Data are represented as mean ± SD. ****P < 0.000 1; one-way ANOVA (**c**); Pearson’s *R* correlation (**d**)

Oral cancer, one of the subtypes of head and neck cancer, has typical hypoxic characteristics and abnormal EGFR activation^16,21^. We first examined whether hypoxia alone can induce EGFR phosphorylation in oral cancer cells. To this end, four oral cancer cell lines (CAL-27, HN4, HN6, and HN30) were subjected to serum starvation overnight and subsequently cultured in serum-free medium under normoxic or hypoxic conditions for 48 h (Fig. 1a and Supplementary Fig. S1a–S1c). Compared with normoxic conditions, hypoxic conditions caused EGFR phosphorylation in all of these tumor cell lines to various extents, suggesting that hypoxia is sufficient to induce EGFR phosphorylation in the absence of EGFR ligands. Unlike ligand binding, which resulted in rapid (occurring in 5 min) and transient (peaking within 30 min) EGFR phosphorylation (Supplementary Fig. S1d), hypoxia induced chronic (apparent after 12 h of hypoxic culture) but constitutive (over the course of 48 h) EGFR phosphorylation at multiple tyrosine residues (Y1045, Y1068, Y1086, and Y845). Crosslinking assays were conducted to evaluate the dimerization status of EGFR, an initiating event in EGFR activation. As shown in Supplementary Fig. S1e, compared to normoxic culture, hypoxic culture significantly increased EGFR dimerization. Given that EGFR mutations may also lead to spontaneous autophosphorylation, we tested the common activating mutations (L858R, exon 19 deletion, T790M, and G719X in exons 18-21) in these cell lines^22,23^. All four cell lines harbored wt EGFR (Supplementary Fig. S1f and Supplementary Table S1), thus ruling out the possible impact of mutations on EGFR activation.

These results indicate that hypoxia can promote EGFR dimerization and induce noncanonical ligand-independent EGFR phosphorylation distinct from that mediated by ligand binding.

### Lysosomal degradation attenuates hypoxia-induced constitutive EGFR phosphorylation

**Fig. 2:**
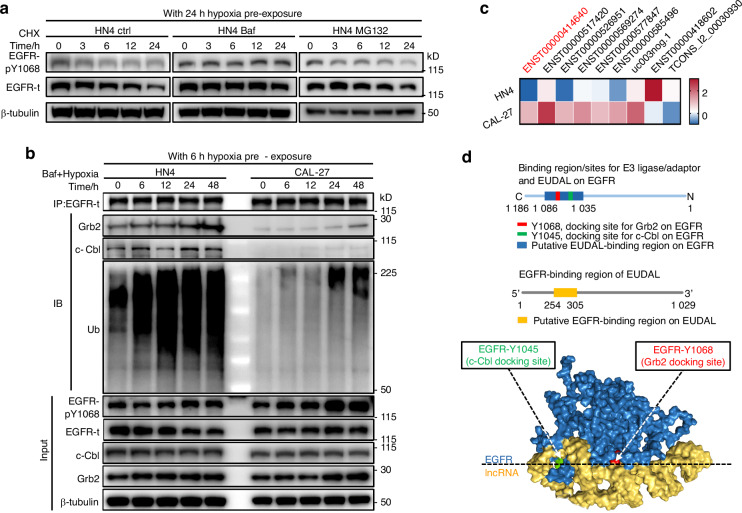
Hypoxia-induced EGFR activation correlates with reduced lysosomal degradation. **a** Representative immunoblot images and half-life analysis of pEGFR and total EGFR levels of HN4 cells after CHX treatment in the absence and presence of 40 nM Bafilomycin A1 (Baf) or 5 μM MG132. **b** A time-course analysis of EGFR ubiquitination and the interaction between EGFR and c-Cbl, as well as Grb2, in hypoxic HN4 and CAL-27 cells. **c** RIP-seq analysis identified a panel of pEGFR-associated lncRNAs. **d** The putative binding region of EUDAL (yellow) on EGFR (blue) covers the docking sites for c-Cbl (green) and Grb2 (red) on EGFR. Data were from representative results of at least three independent experiments

Intriguingly, although similar degrees of EGFR dimerization and levels of EGFR phosphorylation were observed in all tumor cell lines within the first 6 h of exposure to hypoxia, the level of pEGFR did not remain constant in these cells during the subsequent 48-h observation period. A sustained increase in EGFR phosphorylation was detected in CAL-27 and HN30 cells throughout the hypoxic culture period, while EGFR phosphorylation was maintained at low levels in HN4 and HN6 cells (Fig. 1a and Supplementary Fig. S1a, b). These reproducible results prompted us to ask why tumor cells exhibit different responses to hypoxia-induced EGFR activation. Endocytic internalization and sorting have been linked to the strength and duration of EGFR signaling^24,25^. Upon its phosphorylation, EGFR undergoes endocytosis, after which it can be sorted into lysosomes for degradation, which impairs EGFR activation, or be recycled to the cell surface to maintain EGFR signaling. We therefore investigated the intracellular trafficking of pEGFR in hypoxic cells with sustained high or low levels of pEGFR. Analysis of the endocytosis rate revealed that more pEGFR was internalized by HN4 and HN6 cells than by CAL-27 and HN30 cells (Fig. 1b). Accordingly, flow cytometric analysis and confocal imaging showed that more EGFR/pEGFR remained on the cell surface in CAL-27 and HN30 cells than in HN4 and HN6 cells (Fig. 1c, d and Supplementary Fig. S2a, b). These results indicate that more EGFR enters HN4 and HN6 cells after hypoxia-induced phosphorylation. The intracellular distribution of internalized pEGFR was then investigated. pEGFR was colocalized with LAMP1 (a lysosomal marker) but not with RAB11 (the master regulator of the recycling pathway) in hypoxic HN4 and HN6 cells, suggesting that intracellular EGFR was directed to lysosomes but not routed to recycling pathways (Fig. 1d and Supplementary Fig. S2a, b). Next, the half-life of pEGFR was measured in hypoxia-precultured tumor cells in the presence of CHX (Fig. 1e and Supplementary Fig. S2c, d). After an additional 24 h of hypoxic culture, the abundance of pEGFR in HN4 and HN6 cells was decreased by ~70%. Conversely, the level of pEGFR remained stable (degradation rate of <20%) in CAL-27 and HN30 cells (Fig. 1e, Supplementary Fig. S2c, d and Supplementary Table S2). Then, HN4 and HN6 cells were treated with the lysosome inhibitor bafilomycin A1 (Baf) or the proteasome inhibitor MG132 (Fig. 2a, Supplementary Fig. S2e, f, and Supplementary Table S2). EGFR phosphorylation increased upon treatment with Baf but not with MG132, confirming that the degradation of pEGFR in HN4 and HN6 cells is mediated through the lysosomal pathway. These results demonstrate that the lysosomal degradation of internalized pEGFR attenuates hypoxia-induced constitutive EGFR phosphorylation in HN4 and HN6 cells. Conversely, pEGFR remains stable in the cell membrane in CAL-27 and HN30 cells (Fig. 1b–e and Supplementary Fig. S2a–d), thereby prolonging EGFR signaling.

### Binding of EUDAL to EGFR prevents its ubiquitination, endocytosis, and lysosomal degradation

c-Cbl and Cbl-b are recruited to phosphorylated EGFR, leading to its ubiquitination, which is critical for EGFR endocytosis and lysosomal sorting^24–27^. A time course analysis of EGFR ubiquitination and the interaction between EGFR and both c-Cbl and Grb2 was performed (Fig. 2b and Supplementary Fig. S3a). c-Cbl and Grb2 interacted strongly with EGFR in HN4 and HN6 cells, accompanied by obvious ubiquitination of EGFR. In sharp contrast, weak interactions between EGFR and c-Cbl/Grb2, as well as lower levels of EGFR ubiquitination, were observed in hypoxic CAL-27 and HN30 cells.

**Fig. 3:**
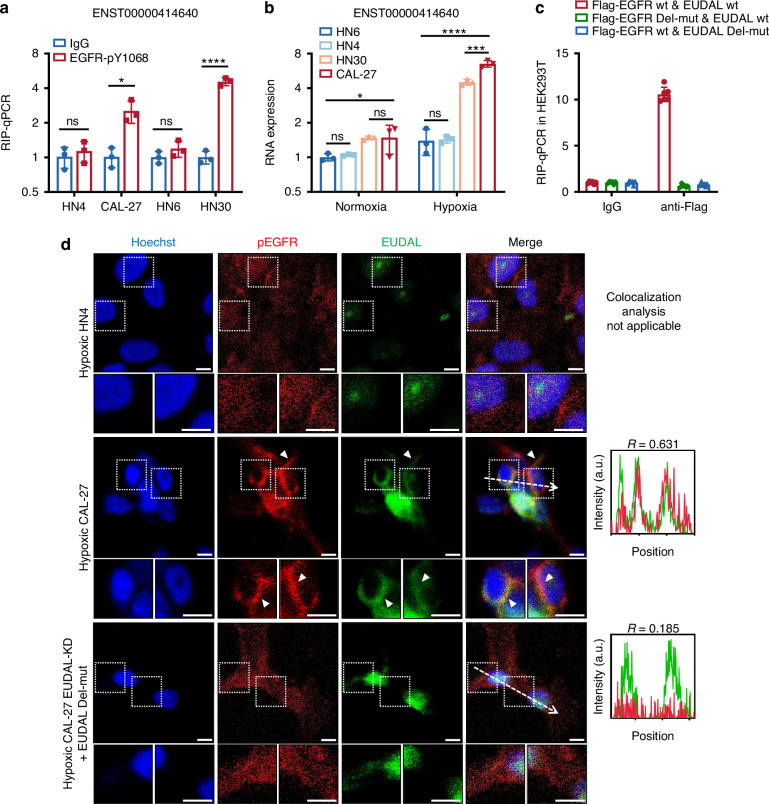
EUDAL directly interacts with EGFR in cells with sustained high levels of EGFR activation. **a** The enrichment of EUDAL in EGFR immunoprecipitates from CAL-27 and HN30 cells (*n* = 3). **b** Higher expression of EUDAL in CAL-27 and HN30 cells (*n* = 3). **c** RIP-qPCR results showed that EUDAL Del-mut and EGFR Del-mut lost the RNA-protein binding capacity. **d** The colocalization of pEGFR and EUDAL in hypoxic HN4 and CAL-27 cells following indicated treatments, as shown by confocal microscopy images. Representative colocalization areas were pointed with arrowheads. The colocalization of pEGFR (red) and EUDAL (green) was estimated with plot profile using ImageJ. Correlation coefficient *R* > 0.6 was considered well-collocated, while *R* < 0.6 was taken as poorly collocated. Bar, 10 µm. Data were mean values of at least three independent experiments. Data are represented as mean ± SD. ns no significance; *P < 0.05; ***P < 0.001; ****P < 0.000 1; unpaired Student’s *t*-test (**a**); one-way ANOVA with Turkey’s Honestly Significant Difference test (**b**); Pearson’s *R* correlation (**d**). wt wild-type, EUDAL Del-mut a lncRNA mutant with an EGFR-binding motif deletion (Δ254–305 nt), EGFR Del-mut an EGFR deletion mutant lacking the binding region (residues 1 035–1 086)

Then RNA immunoprecipitation sequencing with an antibody against EGFR-pY1068 was performed in hypoxic CAL-27 and HN4 cells, respectively. A total of 7 lncRNAs were enriched in CAL-27 cells compared to HN4 cells (fold change ≥ 2; Fig. 2c). Among them, the lncRNA ENST00000414640 attracted our interest because bioinformatics analysis revealed that the putative binding region of this lncRNA on EGFR (residues 1 035–1 086) covers the docking sites for both c-Cbl (residue 1045) and Grb2 (residue 1 068) on EGFR, suggesting that this lncRNA may compete with the c-Cbl/Grb2 complex for binding to EGFR (Fig. 2d). We thus named it EGFR ubiquitination- and degradation-associated lncRNA (EUDAL). The enrichment of EUDAL in the EGFR immunoprecipitates was validated by RIP-qPCR (Fig. 3a). Greater expression of EUDAL was also found in CAL-27 and HN30 cells than in HN4 and HN6 cells, and this difference in expression was more pronounced under hypoxic conditions (Fig. 3b). RNA pull-down assays showed that only the sense chain of EUDAL but not its antisense chain pulled down the EGFR protein in CAL-27 cell lysates as well as in a cell-free system (Supplementary Fig. S3b). A mutant lncRNA with an EGFR-binding motif deletion (Δ254–305 nt) (EUDAL Del-mut) and an EGFR deletion mutant lacking the EUDAL-binding region (Δ1 035–1 086 aa) (EGFR Del-mut) were constructed and used for RNA pull-down and RIP–qPCR experiments. These mutants no longer exhibited mutual binding capacity (Fig. 3c, Supplementary Fig. S3b and S3d–f). RNA fluorescence in situ hybridization (FISH) and confocal microscopy indicated a high expression level of EUDAL and a high degree of colocalization with pEGFR on the plasma membrane in hypoxia-treated CAL-27 and HN30 cells. In contrast, low levels of EUDAL were observed in HN4 and HN6 cells (Fig. 3d and Supplementary Fig. S3c). Forced expression of EUDAL Del-mut in CAL-27 and HN30 cells with silencing of endogenous EUDAL (CAL-27 EUDAL Del-mut and HN30 EUDAL Del-mut cells) abolished the colocalization of lncRNA and EGFR (Fig. 3d and Supplementary Fig. S3c). Collectively, these findings indicate the existence of a direct interaction between EUDAL and EGFR.

**Fig. 4:**
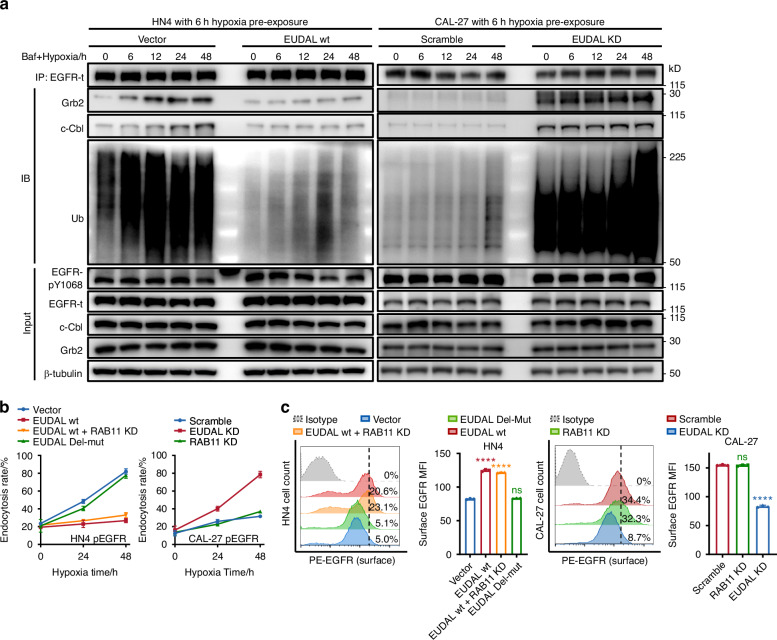
Binding of EUDAL to EGFR prevents its ubiquitination and endocytosis. **a** Left: The ectopic expression of EUDAL wt blocked the interaction between c-Cbl/Grb2 and EGFR, and attenuated subsequent ubiquitination in hypoxic HN4 cells. Right: Silencing of EUDAL promoted binding of c-Cbl/Grb2 to EGFR and elevated EGFR ubiquitination levels in hypoxic CAL-27 cells (*n* = 3). **b** pEGFR endocytosis rate in hypoxic HN4 and CAL-27 following indicated treatments. **c** Flow cytometric analysis of membrane-localized EGFR on hypoxic HN4 and CAL-27 cells (*n* = 3). Data are represented as mean ± SD. ns no significance; ****Endocytosis rate % < 0.000 1; one-way ANOVA with Turkey’s Honestly Significant Difference test (**c**). Baf Bafilomycin A1, wt wild-type, EUDAL Del-mut a lncRNA mutant with an EGFR-binding motif deletion (Δ254–305 nt); KD knockdown

**Fig. 5:**
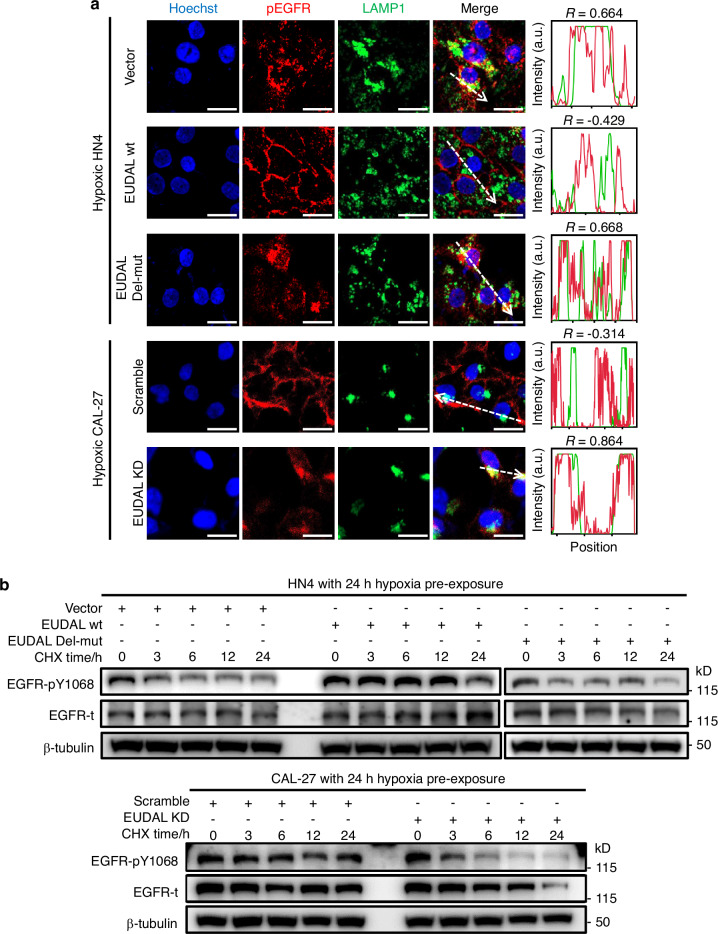
Binding of EUDAL to EGFR prevents its endocytosis and lysosomal degradation. **a** The colocalization of pEGFR with the lysosome marker LAMP1 in hypoxic HN4 and CAL-27 cells following indicated treatments, as shown by confocal microscopy images. The colocalization of pEGFR (red) and LAMP1 (green) was estimated with plot profile using ImageJ. Correlation coefficient *R* > 0.6 was considered well-collocated, while *R* < 0.6 was taken as poorly collocated. Bar, 20 µm. Percentage of cells with high EGFR intensity was indicated. **b** Representative immunoblot images of pEGFR and total EGFR levels after CHX treatment following the indicated treatments. Data were from representative results of at least three independent experiments. Pearson’s *R* (**a**). Baf Bafilomycin A1, wt wild-type, EUDAL Del-mut a lncRNA mutant with an EGFR-binding motif deletion (Δ254–305 nt), KD knockdown

To explore whether EUDAL binding affects the intracellular fate of pEGFR, EUDAL wt or EUDAL Del-mut was ectopically expressed in HN4 and HN6 cells (HN4/HN6 EUDAL wt and HN4/HN6 EUDAL Del-mut cells), and endogenous EUDAL was silenced in CAL-27 and HN30 cells (CAL-27/HN30 EUDAL KD cells) (Supplementary Fig. S3g, h). Ectopic expression of EUDAL wt but not of EUDAL Del-mut abolished the interaction between c-Cbl/Grb2 and EGFR, which in turn attenuated subsequent EGFR ubiquitination and endocytosis in both hypoxic HN4 and HN6 cells (Fig. 4a left, Fig. 4b and Supplementary Fig. S4a–c). This led to the translocation of pEGFR from lysosomes to the plasma membrane, prolonging its membrane retention and half-life (Figs. 4c, 5a, b, Supplementary Figs. S4e, f, S5a, b, and Supplementary Table S2). In contrast, silencing of EUDAL restored both the binding of c-Cbl/Grb2 to EGFR and the EGFR ubiquitination level in hypoxic CAL-27 and HN30 cells (Fig. 4a right and Supplementary Fig. S4d), accompanied by enhanced EGFR endocytosis and lysosomal localization, along with reduced plasma membrane retention and half-life of pEGFR (Figs. 4b, c, 5a, b, Supplementary Figs. S4c–f, S5a, b, and Supplementary Table S2). As expected, RAB11 knockdown did not alter the endocytosis rate, cellular localization, or half-life of pEGFR in any of the tumor cell lines (Fig. 4b, c and Supplementary Figs. S4c–f and S5c, d).

These data indicate that under conditions of hypoxia-induced EGFR activation, EUDAL competes with c-Cbl/Grb2 for binding to EGFR, preventing pEGFR ubiquitination, endocytosis, lysosomal sorting, and degradation, thus prolonging EGFR signaling.

### Hypoxia inducible factor-1α (HIF-1α) upregulates EUDAL transcription by binding to hypoxia response elements (HREs) in its promoter region

**Fig. 6:**
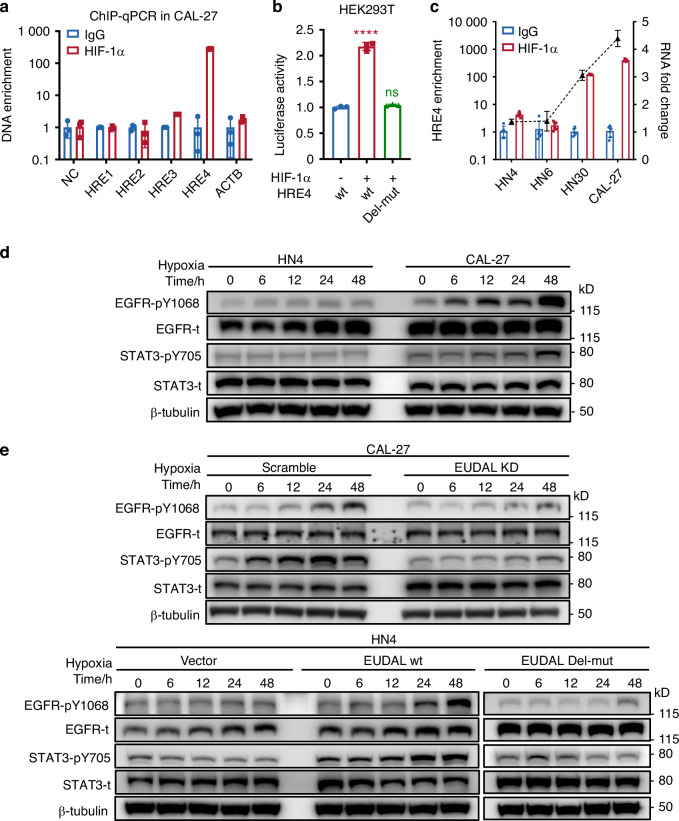
Hypoxia upregulates EUDAL transcription via HIF-1α, induces EGFR phosphorylation and leads to activation of STAT3 signaling. **a** ChIP-qPCR results indicated that HIF-1α precipitants showed the occupancy of HIF-1α on HRE4 (*n* = 3). **b** Luciferase reporter assays showed HIF-1α could directly activate EUDAL transcription (*n* = 3). **c** ChIP-qPCR showed a higher combination of HIF-1α on HRE4 in CAL-27 and HN30 (bars, left *y*-axis), which coincided with higher expression fold-change in these cells (dotted line, right *y*-axis). **d** Representative immunoblot images of indicated protein levels. **e** Upper: Knockdown of EUDAL in CAL-27 cells inhibited phosphorylation of both EGFR and STAT3. Lower: Hypoxia treatment combined with ectopic expression of EUDAL can simultaneously lead to phosphorylation of EGFR and STAT3 in HN4 cells. Data were from representative results of at least three independent experiments. Data are represented as mean ± SD. ns no significance; ****P < 0.000 1; one-way ANOVA with Turkey’s Honestly Significant Difference test (**b**). HRE4 Del-mut, a deletion mutant lacking the −525 to −551 bp segment in the EUDAL promoter region; wt wild-type, EUDAL Del-mut an lncRNA mutant with an EGFR-binding motif deletion (Δ254–305 nt)

Previously, we found that compared with normoxic cells, hypoxic CAL-27 and HN30 cells exhibited significantly increased EUDAL levels, suggesting the potential impact of hypoxia on EUDAL expression (Fig. 3b). HIF-1α is one of the key transcription factors that activate downstream targets within tumor cells in response to hypoxia^28^. We sought to determine whether EUDAL transcription is regulated directly by HIF-1α. To this end, HIF-1α was knocked down in CAL-27 and HN30 cells (Supplementary Fig. S6a). Under hypoxic conditions, HIF-1α KD dramatically reduced the expression of EUDAL and the phosphorylation of EGFR (Supplementary Fig. S6b, c). Then, four putative HREs in the promoter of EUDAL were predicted by in silico analysis (Supplementary Fig. S6d). qPCR primers corresponding to these HREs (HRE1-4) and a non-HRE (NC) region of the EUDAL promoter were designed (Supplementary Fig. S6e and Supplementary Table S3). The crosslinked HIF-1α-chromatin complexes were immunoprecipitated with an anti-HIF-1α antibody in hypoxic CAL-27. Chromatin immunoprecipitation (ChIP)-qPCR showed the occupancy of HIF-1α on HRE4 under hypoxia, indicating that the specific HIF-1α binding site in the EUDAL promoter is located at −525 to −551 bp upstream of the transcription start site (Fig. 6a and Supplementary Fig. S6f). The results of the reverse ChIP assay also indicated that HIF-1α can bind to the promoter region of EUDAL (Supplementary Fig. S6g). In addition, luciferase reporter assays were applied to test whether HIF-1α can directly activate EUDAL transcription. Luciferase reporter constructs containing the full-length EUDAL promoter region (HRE4 wt) or the EUDAL promoter with deletion of bp −525 to −551 (HRE4 Del-mut) were cotransfected with the HIF-1α expression plasmid. As shown in Fig. 6b, the luciferase activity of the HRE4 wt construct but not the HRE4 Del-mut construct was markedly increased in HIF-1α-transfected cells.

Compared to the pronounced induction of EUDAL expression by hypoxia in CAL-27 and HN30 cells (3.06–4.39-fold), only modest upregulation was observed in HN4 and HN6 cells under hypoxic conditions (1.37–1.39-fold) (Fig. 3b). Therefore, a ChIP assay was performed to measure HIF-1α occupancy at HRE4 in four cell lines. Higher HIF-1α enrichment at HRE4 was observed in CAL-27 and HN30 (Fig. 6c), suggesting that CAL-27 and HN30 cells have a more accessible chromatin state at the EUDAL promoter, which facilitates HIF-1α-mediated transcriptional activation of EUDAL under hypoxia.

These results demonstrated that a hypoxic environment not only induces the phosphorylation of EGFR in tumor cells but also promotes the transcriptional activity of EUDAL, which is conducive to maintaining sustained high levels of EGFR phosphorylation.

### Hypoxia-induced sustained EGFR signaling endows tumor cells with drug resistance through STAT3-mediated induction of autophagy

Unlike those of ligand-stimulated EGFR activation, the downstream signals of noncanonical EGFR activation and their biological significance have never been clearly described. Akt and ERK are major downstream signaling molecules involved in the biological regulation of ligand-stimulated EGFR activation, and STATs (STAT1 and STAT3) reportedly participate in the stress-induced EGFR pathway^29–31^. Thus, the activation status of these effectors was evaluated. As shown in Fig. 6d, Supplementary Fig. S7a, b, and Supplementary Table S2, the activation/phosphorylation of STAT3 rather than the canonical signaling molecules (Akt and ERK) or STAT1 occurred in hypoxic CAL-27 and HN30 cells. Then, ectopic expression of EUDAL wt or EUDAL Del-mut in HN4 and HN6 cells, as well as knockdown of EUDAL in CAL-27 and HN30 cells, was conducted. Hypoxia alone was not sufficient to promote STAT3 activation in HN4 and HN6 cells. However, hypoxia combined with ectopic expression of EUDAL wt but not EUDAL Del-mut led to simultaneous phosphorylation of EGFR and STAT3. In contrast, knockdown of EUDAL in CAL-27 and HN30 cells inhibited the phosphorylation of both EGFR and STAT3 (Fig. 6e, Supplementary Fig. S7c, d, and Supplementary Table S2).

**Fig. 7:**
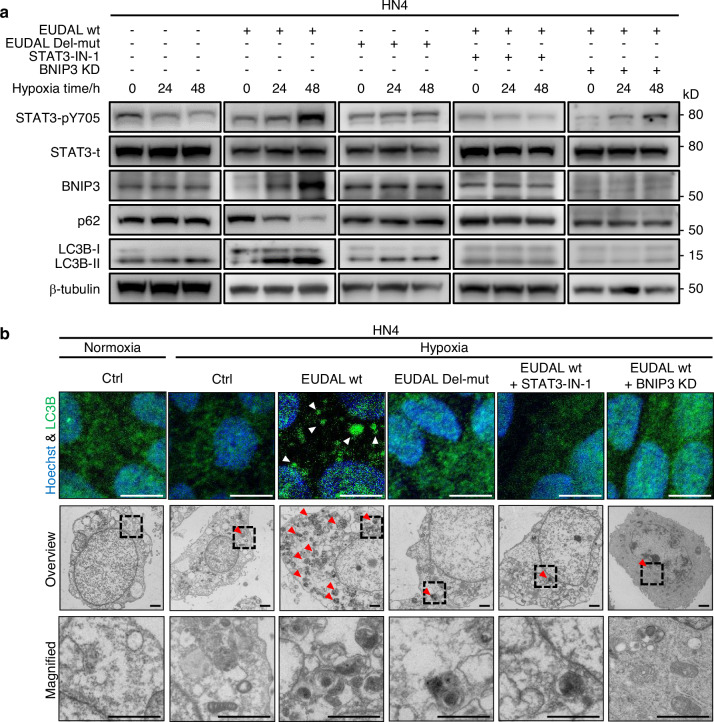
Hypoxia/EUDAL/STAT3 axis activation results in elevated autophagic flux in tumor cells with originally lower pEGFR levels. **a** Expression analysis of autophagy-related markers LC3B and p62, as well as pSTAT3 and BNIP3, in HN4 cells following indicated treatments. **b** Upper: LC3B puncta (white arrowed) identified by fluorescence microscopy in HN4 cells following the indicated treatments. Bar for fluorescent microscopies, 10 μm. Lower: Examination of autophagosome (red arrowed) in HN4 cells following indicated treatments by transmission electron microscopy. Bar for electronic microscopies, 1 μm. Data were from representative results of at least three independent experiments. wt wild-type; EUDAL Del-mut, a lncRNA mutant with an EGFR-binding motif deletion (Δ254–305 nt); KD knockdown

**Fig. 8:**
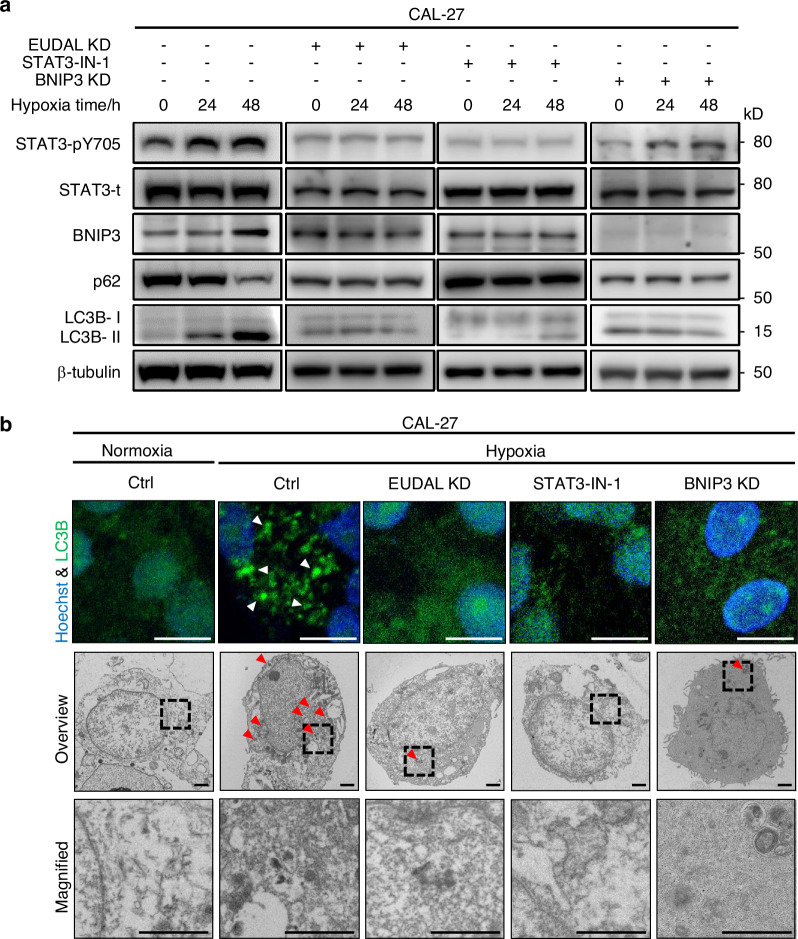
Hypoxia/EUDAL/STAT3 axis activation results in elevated autophagic flux in tumor cells with originally higher pEGFR levels. **a** Expression analysis of autophagy-related markers LC3B and p62, as well as pSTAT3 and BNIP3, in CAL-27 cells following indicated treatments. **b** Upper: LC3B puncta (white arrowed) identified by fluorescence microscopy in CAL-27 cells following the indicated treatments. Bar for fluorescent microscopies, 10 μm. Lower: Examination of autophagosome (red arrowed) in CAL-27 cells following indicated treatments by transmission electron microscopy. Bar for electronic microscopies, 1 μm. Data were from representative results of at least three independent experiments. wt wild-type, EUDAL Del-mut a lncRNA mutant with an EGFR-binding motif deletion (Δ254–305 nt), KD knockdown

Recent evidence has highlighted a novel function of STAT3 in autophagy induction under hypoxic conditions through multiple downstream effectors, such as BNIP3, ATF4, and ATF6^32–35^. In line with these reports, a time-dependent increase in LC3B-II (a marker of autophagy) levels, accumulation of LC3B puncta, presence of autophagosomes, increase in BNIP3 expression, and degradation of the autophagy substrate p62 were observed in hypoxic CAL-27 and HN30 cells but not in HN4 and HN6 cells (Figs. 7, 8, Supplementary Figs. S7e, f, S8 and S9). EUDAL wt promoted autophagy in hypoxic HN4 and HN6 cells. Conversely, EUDAL silencing, STAT3-IN-1 (a STAT3 inhibitor) treatment, or BNIP3 silencing suppressed autophagic flux in hypoxic CAL-27 and HN30 cells or EUDAL wt-overexpressed HN4 and HN6 cells (Figs. 7, 8 and Supplementary Figs. S8, S9). These results indicate that hypoxia-induced EUDAL/EGFR/STAT3/BNIP3 signaling is sufficient and necessary to induce autophagy in hypoxic tumor cells.

We then tested the effect of hypoxia on the biological behavior of tumor cells. No significant changes in cell proliferation, colony formation, apoptosis, or migration were observed in the hypoxic cultures compared with the corresponding normoxic controls (Supplementary Fig. S10a–e). Compared with normoxic conditions, hypoxia treatment induced resistance to cisplatin (the IC_50_ increased by 3.25–3.67-fold) in CAL-27 and HN30 cells, but had little effect on HN4 and HN6 cells (Supplementary Fig. S11a).

**Fig. 9:**
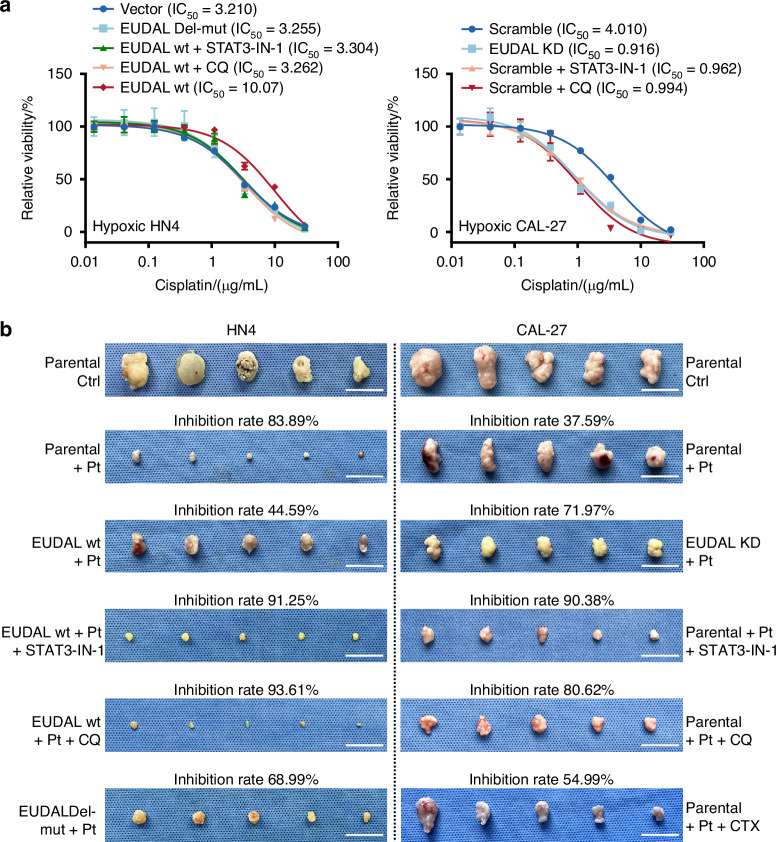
Hypoxia confers tumor cells drug resistance through STAT3-mediated induction of autophagy. **a** IC_50_ values for cisplatin were determined in hypoxic HN4 and CAL-27 receiving indicated treatments. **b** Tumor-bearing mice models were established by inoculating the indicated cell lines. Xenografts were treated with cisplatin alone or in combination with STAT3-IN-1, CQ or cetuximab. Tumor inhibition rates were determined in each experimental group. *n* = 5 for IC_50_ estimation, *n* = 5 for in vivo animal experiments. Data are represented as mean ± SD. CTX cetuximab, Pt cisplatin, wt wild-type, EUDAL Del-mut a lncRNA mutant with an EGFR-binding motif deletion (Δ254–305 nt), KD knockdown

Autophagy is a cellular catabolic process that plays a cytoprotective role in tumors, especially in promoting drug resistance^36^. Hence, we speculate that EUDAL/EGFR/STAT3 signaling-induced autophagy may lead to drug resistance in these tumor cells. Compared with EUDAL Del-mut, ectopic expression of EUDAL wt led to resistance to cisplatin (the IC_50_ increased by 3.14- and 3.11-fold) in hypoxic HN4 and HN6 cells, and further treatment with STAT3-IN-1 or CQ (an autophagy inhibitor) restored the low IC_50_ value (Fig. 9a and Supplementary Fig. S11b). Conversely, silencing of EUDAL or treatment with STAT3-IN-1 or CQ reduced the IC_50_ values in hypoxic CAL-27 (a decrease of 4.03–4.38-fold) and HN30 (a decrease of 2.25–2.45-fold) cells to values close to or even lower than those in normoxic culture (Fig. 9a and Supplementary Fig. S11b).

Collectively, these data show that hypoxia-induced sustained EGFR activation endows tumor cells with drug resistance through STAT3-mediated induction of autophagy.

### Effects of EUDAL, STAT3 activation, and autophagy on drug resistance in vivo

To confirm that the induction of autophagy via EUDAL and STAT3 affects drug resistance in vivo, tumor-bearing mouse models were established by inoculating parental HN4/HN6, HN4/HN6 EUDAL wt, HN4/HN6 EUDAL Del-mut, parental CAL-27/HN30, and CAL-27/HN30 EUDAL KD cells. The xenograft-bearing mice were then treated with cisplatin alone or in combination with STAT3-IN-1, CQ or anti-EGFR antibody cetuximab (Fig. 9b, Supplementary Figs. S11c, d and S12a). As expected, the administration of cisplatin resulted in significant tumor regression, with inhibition rates of approximately 80.37%–83.89% in parental HN4 and HN6 cell-bearing mice. Treatment with EUDAL wt but not with EUDAL Del-mut significantly impaired the antitumor effects of cisplatin (the tumor inhibition rate decreased to approximately 44.59%–45.74%). Treatment with STAT3-IN-1 and CQ restored the sensitivity of HN4 and HN6 EUDAL wt cells to cisplatin. Compared with parental CAL-27 and HN30 xenograft-bearing mice (with tumor inhibition rates of 37.59%–48.43%), EUDAL KD xenograft-bearing mice displayed better tumor inhibition effects when treated with cisplatin (with tumor inhibition rates of 71.97%–78.63%). Enhanced antitumor responses were also observed in parental CAL-27 and HN30 xenograft-bearing mice treated with cisplatin combined with STAT3-IN-1 (with tumor inhibition rates of 84.58%–90.38%) or CQ (with tumor inhibition rates of 80.62%–86.25%). However, the combined treatment with cisplatin and cetuximab failed to display additive antitumor effects in all four cell lines in vivo or in vitro (Fig. 9b, Supplementary Fig. S11c, d, and S12b, c). This outcome is not unexpected, since EGFR monoclonal antibodies are clinically indicated for EGFR-mutated cells rather than wild-type cells, as utilized in the present study^11,12,37^. Immunohistochemical analysis revealed elevated LC3B levels in animal tumors bearing EUDAL wild-type (wt) cells with or without cisplatin treatment (Supplementary Fig. S12a). EUDAL silencing or STAT3 inhibitor treatment attenuated LC3B levels. Treatment with CQ increased LC3B levels in tumor tissues. This effect occurs because CQ suppresses autophagy by inhibiting lysosomal acidification, consequently causing accumulation of inactive autophagosomes containing LC3B^38^.

These results indicate that EUDAL/EGFR/STAT3/BNIP3 signaling and subsequent autophagy induction play essential roles in drug resistance in vivo. Blockade of this axis could thus sensitize tumor cells to chemotherapy.

### The EUDAL/EGFR/STAT3/autophagy signaling pathway predicts response to chemotherapy in patients with oral cancer

**Fig. 10:**
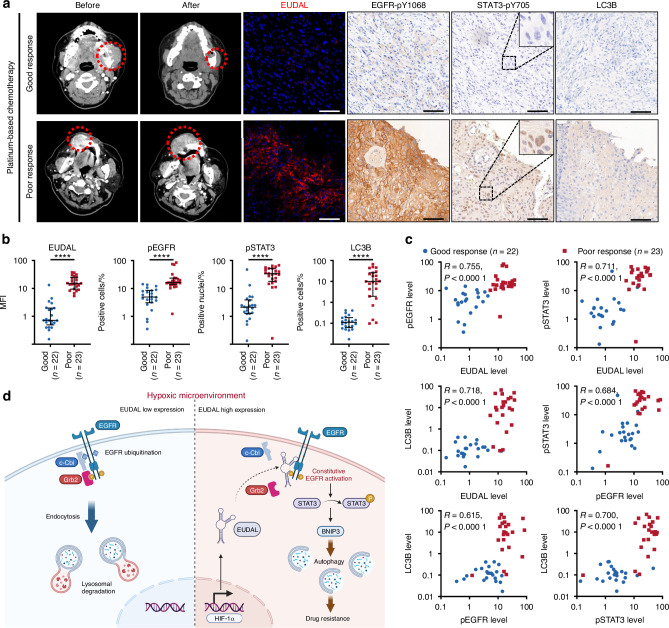
EUDAL/EGFR/STAT3/autophagy signal axis reflects the efficacy of chemotherapy in patients with oral cancer. **a** Left: Representative CT scans in patients having a good response or a poor response before and after receiving platinum-based chemotherapy. Right: Representative immunohistochemistry staining for pEGFR, pSTAT3, and LC3B, and RNA FISH staining for EUDAL in tumor tissues from patients with a good or a poor response to Platinum-based chemotherapy. **b** Semi-quantitative analysis showed lower EUDAL, pEGFR, pSTAT3, and LC3B levels in good responders than in poor responders. Error bars: mean with interquartile range. **c** Correlations among EUDAL, pEGFR, pSTAT3, and LC3B levels in tumor tissues. **d** A proposal schematic model illustrating that a novel lncRNA EUDAL facilitates the hypoxia-induced noncanonical phosphorylation of wild-type EGFR and drug resistance by competitively binding to EGFR with c-Cbl and Grb2. *n* = 45. ****P < 0.000 1; Mann–Whitney *U* test (**b**), Spearman rank correlation (**c**)

Next, we evaluated the potential relationships between the levels of EUDAL, pEGFR, and pSTAT3 in oral cancer patients and the drug response in these patients. Forty-five patients who received platinum-based chemotherapy were included in our analysis (Supplementary Table S4). Twenty-two patients had a clinical/imaging-confirmed complete response (CR) or partial response (PR), and 23 patients had clinical/imaging-confirmed stable disease (SD) or progressive disease (PD). In this study, patients with CR or PR were considered good responders, while those with SD or PD were considered poor responders (Fig. 10a). The levels of EUDAL, pEGFR, pSTAT3, and LC3B were significantly higher in poor responders than in good responders (Fig. 10a, b, and Supplementary Fig. S12d). Significant correlations were also detected among the levels of EUDAL, pEGFR, pSTAT3, and LC3B (correlation coefficients of 0.615–0.755, *P* < 0.000 1; Fig. 10c).

To rule out the possible influence of chemotherapy on the activation of EUDAL/EGFR/STAT3/autophagy axis, we collected tumor samples from an additional 45 oral cancer patients who did not receive chemotherapy. Similarly, patients with high EUDAL expression exhibited elevated levels of pEGFR, pSTAT3, and LC3B in their tumors (Fig. S12e, f).

These data indicate that the activity of the EUDAL/EGFR/STAT3/autophagy signaling axis may predict the therapeutic efficacy of chemotherapy in patients with oral cancer.

## Discussion

This study revealed for the first time a previously unrecognized direct regulatory effect of hypoxia on ligand-independent constitutive EGFR activation and downstream signaling. The role of EUDAL in shaping the tumor cell response to hypoxia-induced EGFR activation was also elucidated. Hypoxia can promote EGFR dimerization and induce noncanonical EGFR phosphorylation. EUDAL can compete with E3 ligases for binding to EGFR and prevent its ubiquitination, endocytosis, and lysosomal degradation, hence leading to sustained high levels of EGFR phosphorylation and subsequent activation of downstream STAT3 signaling. STAT3-mediated induction of autophagy ultimately endows tumor cells with resistance to chemotherapy (Fig. 10d).

Dysregulation of EGFR and its downstream signaling pathways has long been considered essential regulators of cancer progression^1,39^. Although the role of EGFR is best characterized in the context of ligand-dependent activation, accumulating evidence shows that EGFR signaling can also be constitutively activated in a ligand-independent manner^40^. Receptor mutations are generally considered one of the main causes of noncanonical receptor activation. Single point mutations in exon 21 and deletions in exon 19 can result in constitutive activation of EGFR^6,41^. However, Sun et al. evaluated 32 common tumor types and reported that EGFR exhibited a surprisingly low mutation frequency (overall mutation frequency, 2.8%)^42^. For head and neck cancer, only <5% of tumors contain EGFR mutations^43^. These results suggest that only a small portion of constitutive EGFR activation in head and neck cancer may be due to mutation-driven activation. Recent observations on constitutive EGFR activation in tumors without mutations further supported this speculation^44,45^. Therefore, several previously unrecognized mechanisms in the tumor microenvironment may greatly contribute to the constitutive activation of EGFR.

Hypoxia is believed to be a common feature of solid tumors. Bhandari et al. quantified hypoxia in 19 types of tumors and reported that head and neck, cervical, and lung cancers were the most hypoxic, while adenocarcinomas of the thyroid and prostate cancer were the least hypoxic^16^. Hypoxia plays a crucial and complex role in tumor physiology and progression, affecting processes ranging from proliferation and angiogenesis to cell death/apoptosis and metastatic spread^28,46^. The crosstalk between hypoxia and EGFR signaling has attracted increasing attention in cancer research. Emerging evidence has demonstrated that tumor hypoxia can increase EGFR expression by promoting its transcription and protein synthesis^17,47^. However, knowledge regarding the causal relationships between tumor hypoxia and noncanonical EGFR activation is limited. Wang et al. reported that hypoxia can promote ligand-independent EGFR signaling activation in clear cell renal cell carcinoma. However, due to the lack of detection of EGFR mutations in tumor cell lines, the possible impact of mutation-induced spontaneous receptor activation could not be excluded in that study^48^.

To our knowledge, our study for the first time systematically elucidates the impact of hypoxia on ligand-independent EGFR phosphorylation and the endocytosis, intracellular fate, and downstream signaling of EGFR in human cancer cells harboring wt EGFR, thus revealing a functional link between these two characteristic events in tumor progression. Our findings showed that hypoxia is sufficient to induce the dimerization and chronic but constitutive phosphorylation of wt EGFR, which is distinct from phosphorylation stimulated by ligand binding. Intriguingly, we found that human oral cancer cells exhibit different EGFR phosphorylation responses to hypoxia. Sustained increases in and high levels of EGFR phosphorylation were detected in some hypoxic tumor cell lines, while EGFR phosphorylation remained low in others. EGFR ubiquitination drives its endocytosis and allows the recognition of internalized EGFR by multivesicular bodies (MVBs), which is essential for its lysosomal degradation^27^. Consistent with these reports, we observed obvious c-Cbl/Grb2-dependent EGFR ubiquitination in tumor cells exhibiting low levels of hypoxia-induced EGFR activation (HN4 and HN6 cells). Robust endocytosis, lysosomal localization, and degradation of pEGFR were found in these cells. Unlike the canonical activation pathway, the RAB11-dependent recycling pathway rarely participates in the intracellular trafficking of internalized EGFR upon hypoxia treatment. One possible explanation for this phenomenon is that during ligand-stimulated canonical activation, EGFR is subjected to multiple rounds of activation, and the recycling pathway could compensate for the rapid loss of EGFR from the plasma membrane, thus ensuring sufficient receptor–ligand binding prior to the next activation cycle^49^. However, in the context of hypoxia-induced constitutive EGFR activation, the EGFR signaling output seems to be controlled mainly by lysosomal degradation, which can prevent prolonged and excessive signaling activation, thus serving as a negative feedback regulatory mechanism to maintain cell homeostasis. Notably, hypoxic tumor cells with constitutive and high EGFR activation (CAL-27 and HN30 cells) appear to employ some previously unknown antagonistic mechanisms to bypass the inhibitory effect of ubiquitination-mediated degradation by blocking the binding of Cbl/Grb2 to EGFR.

By comparing pEGFR-binding molecules, we found that an uncharacterized lncRNA, EUDAL, was highly enriched in hypoxic tumor cells with constitutive and high EGFR activation. We showed that EUDAL is a key determinant of the tumor cell response to hypoxia-induced EGFR activation. High expression of EUDAL facilitates sustained phosphorylation of EGFR in hypoxic tumor cells. We demonstrated the following mechanism for the function of EUDAL: EUDAL can competitively bind to EGFR with the c-Cbl/Grb2 complex, thereby preventing the ubiquitination and subsequent lysosomal degradation of EGFR. Moreover, EUDAL was demonstrated to be a direct transcriptional target of HIF-1α and to be upregulated under hypoxic conditions, thus further increasing hypoxia-induced constitutive EGFR activation in tumors.

Although the ability of canonical EGFR activation to exert oncogenic effects through the downstream Akt and ERK pathways has been well described, the downstream noncanonical EGFR signaling pathways and their biological significance remain poorly understood. Our results indicate that hypoxia-induced EGFR phosphorylation does not trigger Akt or ERK activation but instead leads to the activation of STAT3, which in turn induces autophagy in tumor cells. Our in vitro and in vivo results showed that hypoxic tumor cells with high levels of EGFR activation and autophagy were more resistant to chemotherapy than those with low levels of activated EGFR or autophagy. This result is not surprising, as autophagy is an evolutionarily conserved mechanism for cell survival that can be hijacked by tumor cells to avoid cell death and cause drug resistance^36,50,51^. More importantly, our study also showed that blockade of EUDAL/EGFR/STAT3/autophagy signaling can sensitize tumor cells to chemotherapy and that the EUDAL/EGFR/STAT3/autophagy signaling status can predict the drug response in patients with oral cancer. Therefore, preventing hypoxia-induced EGFR and its downstream signaling activation might be an effective strategy to enhance the efficacy of chemotherapy.

Overall, we demonstrated that hypoxia could promote EGFR dimerization and induce noncanonical EGFR activation. The accumulation of ubiquitinated EGFR impairs its constitutive activation by promoting receptor internalization and lysosomal degradation. The binding of EUDAL to EGFR prevents its ubiquitination and degradation, facilitates its sustained phosphorylation, and subsequently activates downstream STAT3 signaling, which leads to autophagy-related drug resistance in tumor cells.

## Materials and methods

### Ethics statement

This study was conducted according to the guidelines of the Declaration of Helsinki and approved by the Ethics Committee of Shanghai Ninth People’s Hospital Affiliated to Shanghai Jiao Tong University School of Medicine (Ethics number: SH9H-2022-TK270-1). All animal tumor xenograft experiments were conducted in accordance with the Ministry of Health national guidelines for the housing and care of laboratory animals and approved by the Animal Ethics Committee of Shanghai Ninth People’s Hospital (Ethics number: SH9H-2022-A527-SB).

### Patient specimens

The study included 45 oral cancer patients who received platinum-based chemotherapy in the Department of Oral and Maxillofacial Surgery (Ninth People’s Hospital, Shanghai, China) between 2018 and 2020. All patients signed written informed consent. The efficiency of treatment was evaluated by computed tomography (CT) before and after treatment (2 weeks after the last drug treatment). Tumor samples were obtained from the surgical specimens. Pharmaceutical treatment responses were estimated according to the Response Evaluation Criteria in Solid Tumors (RECIST 1.1). In brief, the disappearance of all lesions and pathological lymph nodes was considered a complete response (CR), lesions that decreased by more than 30% with no new lesions or progression of nontarget lesions were considered partial response (PR), lesions that increased by more than 20% or new lesions or progression of nontargeted lesions were considered progressive disease (PD), and no PRs or no PD patients were considered stable disease (SD). Another cohort of chemotherapy-untreated patients with primary oral cancer was obtained with a commercially available tissue microarray from OUTDO BIOTECH (China). 45 patient samples were included after exclusion of poor-quality tissue samples.

### Cell lines and culture conditions

The human oral cancer cell line CAL-27 was obtained from ATCC, and HN4, HN6, and HN30 cell lines were kindly provided by Prof. Li Mao, University of Maryland Dental School. The authenticity of all the cell lines was checked via short tandem repeat (STR) profiling. *Mycoplasma* contamination was routinely tested using Myco-Lumi Luminescent Mycoplasma Detection Kit (Beyotime Technology, China). According to the experimental requirements, the cells were cultured in DMEM supplemented with or without 10% FBS under normoxic (21% O_2_ with 5% CO_2_) or hypoxic (1% O_2_, 94% N_2_, and 5% CO_2_) conditions as indicated.

### Western blotting

Western blotting (WB) was performed as described previously^52^. The primary antibodies used included total EGFR from Merck Millipore (cat. #06-847, RRID: AB_2096607; MA, USA), phospho-EGFR (Tyr1045, cat. #2237, RRID: AB_331710; Tyr1068, cat. #2236, RRID: AB_331792; Tyr1086, cat. #2220, RRID: AB_823485; Tyr845, cat. #6963, RRID: AB_10839407), RAB11 (cat. #5589, RRID: AB_10693925), Ubiquitin (cat. #58395, RRID: AB_3075532), c-Cbl (cat. #8847, RRID: AB_10860763), Grb2 (cat. #3972, RRID: AB_10693935), HIF-1α (cat. #36169, RRID: AB_2799095), total STAT1 (cat. #9172, RRID: AB_2198300), STAT1-pY701 (cat. #9167, RRID: AB_561284), total STAT3 (cat. #9139, RRID: AB_331757), STAT3-pY705 (cat. #9145, RRID: AB_2491009), total ERK1/2 (cat. #4695, RRID: AB_390779), pERK1/2 (pT202/pY204; cat. #4370, RRID: AB_2315112), total Akt (cat. #4685, RRID: AB_2225340), Akt-pS473 (cat. #4060, RRID: AB_2315049), ATF4 (cat. #T55873, RRID: AB_2936990), ATF6 (cat. #TD6009, RRID: AB_2936895), BNIP3 (cat. #T56771), LC3B (cat. #83506, RRID: AB_2800018), and p62 (cat. #5114, RRID: AB_10624872) from Cell Signaling Technology, and β-tubulin (cat. #M20005, RRID: AB_2920648) from Abmart, used as loading controls. Densitometry was implemented with ImageJ (RRID:SCR_003070).

### EGFR dimerization assay

The EGFR dimerization assay adopted BS3 (cat. #HY-124329A, MCE, China) as a cross-linking agent following the protocols described in literature^53^. In brief, after 6 h of normoxic or hypoxic treatment, the cells were washed and cross-linked. The cell lysates were prepared and analyzed via routine denaturing Western blotting.

### EGFR stability assay

The half-life of pEGFR was assessed via WB after blockade of protein synthesis with 20 mol/L cycloheximide (CHX; cat. #HY-12320, MCE, China) for 6, 12, 18, or 24 h after 24 h of hypoxia pre-exposure according to the protocol adopted from Chakraborty et al.^54^. Densitometry was implemented with ImageJ.

### Flow cytometry analysis

The cells were stained with a PE-conjugated anti-EGFR antibody (BD Biosciences, cat. #555997, RRID: AB_396281). The stained cells were then analyzed using a cytometer (BD FACSCalibur, BD Biosciences, CA, USA). Data processing was done using FlowJo software (RRID:SCR_008520).

### Plasmids and lentivirus

Mammalian plasmids that express full-length EGFR, an EGFR deletion mutant lacking the EUDAL-binding region (residues 1035–1086), wild-type EUDAL, and a lncRNA mutant with an EGFR-binding motif deletion (Δ254–305 nt) were purchased from RiboBio (Guangzhou, China). Lipofectamine 2000 (Invitrogen, CA, USA) and Opti-MEM (Gibco, NY, USA) were used for transient transfection. Lentiviruses expressing wild-type EUDAL or the EUDAL mutant were purchased from RiboBio (Guangzhou, China). Cell infection was carried out with polybrene according to the manufacturer’s instructions.

### RNA interference

Small interfering RNAs targeting RAB11, EGFR, EUDAL, HIF-1α, and BNIP3 were purchased from RiboBio (Guangzhou, China). The siRNA targeting sequences used are listed in Supplementary Table S5. Lipofectamine 2000 (Invitrogen, CA, USA) and Opti-MEM (Gibco, NY, USA) were used as transfection reagents.

### Immunofluorescence and RNA fluorescence in situ hybridization (FISH)

Cells were seeded in glass bottom cell culture dishes, fixed with 4% paraformaldehyde and permeabilized with 0.25% Triton X-100 before they were incubated with primary antibodies (anti-EGFR-pY1068, anti-LAMP1, anti-RAB11, and anti-LC3B; Cell Signaling Technology, MA, USA) at 4 °C overnight and with secondary antibodies (anti-rabbit IgG Alexa Fluor 647 conjugated, cat. #4414, RRID: AB_10693544 or anti-mouse IgG Alexa Fluor 555 conjugated, cat. #4409, RRID: AB_1904022; Cell Signaling Technology, MA, USA) at room temperature for 1 h. Cell nuclei were counterstained with Hoechst (Invitrogen, CA, USA). For RNA FISH, fluorescent probes for lncRNAs were designed and synthesized by RiboBio (Guangzhou, China). Fluorescence images were captured via confocal microscopy (Nikon, Japan). pEGFR endocytosis rate analysis was implemented by calculating the membranous and intracellular pEGFR amount on the indicated time point using ImageJ^55^. Colocation of pEGFR and LAMP1 or EUDAL was quantitatively analyzed with Pearson’s *R* using ImageJ and GraphPad Prism 8.0. *R* > 0.6 was considered as well-collocated, while *R* < 0.6 was taken as poorly-collocated^56^.

### Immunoprecipitation (IP) and ubiquitination assays

To help preserve the protein-interactions and protein-modifications under hypoxia, cells were simultaneously treated with 40 nM bafilomycin A1 (MedChemExpress, China), a lysosome acidification inhibitor during the hypoxia treatment^57^. All cells were pre-exposed to hypoxia for 6 h to achieve similar initial levels of EGFR phosphorylation. Then, at the indicated timepoint, cells were lysed with immunoprecipitation lysis buffer containing 1% SDS (for IP and ubiquitination IB) or no SDS (for co-IP and Grb2/c-Cbl IB). The lysates were incubated with an EGFR antibody and protein A/G magnetic beads (Merck Millipore, MA, USA) overnight. The level of ubiquitinated EGFR and the amount of protein bound to EGFR were determined via Western blotting.

### Quantitative PCR (q-PCR)

The relative RNA concentration was quantified via one-step SYBR Green-based q-PCR. ACTB was used as an internal reference. The primers used are listed in Supplementary Table S3.

### RIP-seq

The RNA-binding protein immunoprecipitation (RIP) process was performed using the Magna RIP Kit (Merck Millipore, MA, USA) according to the manufacturer’s instructions. In brief, the cells were lysed after the indicated treatments. Anti-EGFR-pY1068 (Cell Signaling Technology, cat. #2236, RRID: AB_331792) or anti-Flag (Cell Signaling Technology, cat. #14793, RRID: AB_2572291) antibodies were first incubated with magnetic beads before being added to the cell lysate containing protease and RNase inhibitors, after which the mixture was incubated at 4 °C overnight with end-to-end rotation. The RNA was harvested as instructed, followed by RNA-seq or q-PCR as indicated. For sequencing, the purified RIP product was reverse-transcribed and sequenced with an Illumina HiSeq™2500 system.

The lncRNAs enriched in different cell lines were heat-mapped. RIP-qPCR was subsequently performed to verify the screening results. Those enriched in both cell lines or with a CT value >40 were ruled out.

### Bioinformatic prediction

The interaction between proteins and lncRNAs was predicted by catRAPID (service.tartaglialab.com) and HADDOCK (wenmr.science.uu.nl/haddock2.4/). The putative hypoxia-response element (HRE) motifs on the lncRNA promoter were predicted by JASPAR (jaspar.genereg.net)

### RNA pull-down

RNA pull-down was performed with the Pierce Magnetic RNA‒Protein Pull-Down Kit (Thermo Scientific, MA, USA) according to the manufacturer’s instructions. The RNA was 3’-end-destibiotinylated and incubated with magnetic beads. Subsequently, the RNA-loaded magnetic beads were added to the cell lysates or purified protein and incubated at 4 °C overnight. The proteins were eluted and analyzed via Western blotting.

### Chromatin immunoprecipitation-quantitative polymerase chain reaction (ChIP-qPCR)

Chromatin immunoprecipitation (ChIP) was performed as previously described. In brief, the cells were cross-linked with 1% formaldehyde, and the reaction was quenched with glycine. The cells were then lysed. Chromatin solutions were incubated with anti-HIF-1α (Cell Signaling Technology, MA, USA). Protein G magnetic beads were added to capture the immune complexes. The cross-linking reactions were reversed at 65 °C for 2 h, after which the DNA was purified and analyzed via real-time PCR. The primers used in ChIP-qPCR are listed in Table S3.

### Reverse ChIP assay

The reverse ChIP assay was carried out using DNA probes targeting the 2000 bp sequence upstream of the transcription starting site of the *EUDAL* gene with a kit purchased from Bersin Bio (Guangzhou, China). CAL-27 cells were first subjected to hypoxia for 24 h. The reverse ChIP process was implemented strictly according to the manufacturer’s instructions. The beads were washed and finally eluted to obtain the precipitated protein product. Detection of HIF-1α was performed by Western blotting.

### Dual-luciferase reporter assay

HEK293T cells were used as a cell tool. The full-length EUDAL promoter region or the mutant promoter was cotransfected with the HIF-1α expression construct into the pmirGLO vector. The cells were harvested after 24 h of hypoxic culture. Luciferase activity was estimated with a Renilla-Firefly Luciferase Dual Assay Kit (Thermo Scientific, MA, USA).

### Cell viability and the IC_50_ of antitumor drugs

For cell growth estimation, a Cell Counting Kit-8 (CCK-8) was used to estimate cell viability under normoxic or hypoxic conditions. The CCK-8 assay was also used for drug response assessment. In the present study, logarithmic concentrations of cisplatin were used. Dose‒response inhibition calculations were implemented in GraphPad Prism 8.0 to estimate the IC_50_ of each cell line after different treatments.

### In vivo tumor xenograft experiments

Tumor xenograft experiments and maximal tumor size were approved by the Animal Ethics Committee of Shanghai Ninth People’s Hospital. Four-week-old female BALB/c nude mice were obtained from the Shanghai Laboratory Animal Center (Shanghai, China). Approximately 2 × 10^6^ HN4/HN6 cell variants (parental, EUDAL wt-overexpressing, and EUDAL Del-mut-overexpressing) or CAL-27/HN30 cell variants (parental and EUDAL KD) were injected subcutaneously into the right flank of the mice. Mice were randomly assigned to different treatment groups 7 days after inoculation when subcutaneous tumors were palpable. STAT3-IN-1, a small molecule inhibitor of STAT3 (10 mg/kg per week) were administered intragastrically. Cisplatin (5 mg/kg per week) and the autophagy inhibitor chloroquine (CQ) (50 mg/kg per week) were given by intraperitoneal injection. All the reagents were purchased from MedChemExpress (Shanghai, China). Tumor sizes were measured twice a week with an electronic caliper. Tumor volumes were calculated by the following formula: *V* = length × width^2^ × 0.52. After 4 weeks of treatment, the tumor-bearing mice were sacrificed, and the xenografts were excised for further analysis.

### Immunohistochemistry (IHC) and FISH staining

Immunohistochemical staining and semiquantitative assessment were carried out as previously described in detail^58^. The primary antibodies used were against EGFR-pY1068, STAT3-pY705, or LC3B (Cell Signaling Technology, MA, USA). LncRNA levels in the tumor samples were assessed by FISH probes purchased from Servicebio (Wuhan, China). The staining results were estimated in a semiquantitative manner as previously described^59^.

### Statistical analysis

GraphPad Prism 8.0 (RRID:SCR_002798; GraphPad Software, MA, USA) and SPSS 21.0 (RRID:SCR_002865; IBM, NY, USA) were used to implement statistical analysis. Data are presented as mean with standard deviation (SD) unless otherwise indicated. Student’s *t*-test or one-way ANOVA was used to compare means of two or more groups. Pearson’s *R* or Spearman's rank correlation was used for correlation analysis of data with normal or non-normal distribution. The Pearson Chi-square test was used for categorical data comparison. Detailed methods used for comparisons and correlations were presented in the figure legends. For hypothesis testing,*P* < 0.05 was defined as statistically significant.

## Supplementary information

Supplementary Material

## Data Availability

The data, materials, and methods supporting the findings of this study are available from the corresponding author upon reasonable request.

## References

[1] Kalyankrishna, S. & Grandis, J. R. Epidermal growth factor receptor biology in head and neck cancer. *J. Clin. Oncol.* **24**, 2666–2672 (2006). 16763281 10.1200/JCO.2005.04.8306

[2] Schlessinger, J. Ligand-induced, receptor-mediated dimerization and activation of EGF receptor. *Cell* **110**, 669–672 (2002). 12297041 10.1016/s0092-8674(02)00966-2

[3] Endres, N. F. et al. Conformational coupling across the plasma membrane in activation of the EGF receptor. *Cell* **152**, 543–556 (2013). 23374349 10.1016/j.cell.2012.12.032 PMC3718647

[4] Dittmann, K. et al. Radiation-induced epidermal growth factor receptor nuclear import is linked to activation of DNA-dependent protein kinase. *J. Biol. Chem.* **280**, 31182–31189 (2005). 16000298 10.1074/jbc.M506591200

[5] Zwang, Y. & Yarden, Y. p38 MAP kinase mediates stress-induced internalization of EGFR: implications for cancer chemotherapy. *EMBO J.* **25**, 4195–4206 (2006). 16932740 10.1038/sj.emboj.7601297 PMC1570432

[6] Shan, Y. et al. Oncogenic mutations counteract intrinsic disorder in the EGFR kinase and promote receptor dimerization. *Cell* **149**, 860–870 (2012). 22579287 10.1016/j.cell.2012.02.063

[7] Chakraborty, S. Constitutive and ligand-induced EGFR signalling triggers distinct and mutually exclusive downstream signalling networks. *Nat. Commun.* **5**, 5811 (2014). 25503978 10.1038/ncomms6811 PMC4268886

[8] Gao, J., Ulekleiv, C. H. & Halstensen, T. S. Epidermal growth factor (EGF) receptor-ligand based molecular staging predicts prognosis in head and neck squamous cell carcinoma partly due to deregulated EGF-induced amphiregulin expression. *J. Exp. Clin. Cancer Res.* **35**, 151 (2016). 27669890 10.1186/s13046-016-0422-z PMC5037594

[9] Li, A. et al. Gefitinib sensitization of cisplatin-resistant wild-type EGFR non-small cell lung cancer cells. *J. Cancer Res. Clin. Oncol.* **146**, 1737–1749 (2020). 32342201 10.1007/s00432-020-03228-4 PMC7185832

[10] Benhar, M., Engelberg, D. & Levitzki, A. Cisplatin-induced activation of the EGF receptor. *Oncogene* **21**, 8723–8731 (2002). 12483525 10.1038/sj.onc.1205980

[11] Kobayashi, S. et al. EGFR mutation and resistance of non-small-cell lung cancer to gefitinib. *N. Engl. J. Med.* **352**, 786–792 (2005). 15728811 10.1056/NEJMoa044238

[12] Lynch, T. J. et al. Activating mutations in the epidermal growth factor receptor underlying responsiveness of non-small-cell lung cancer to gefitinib. *N. Engl. J. Med.* **350**, 2129–2139 (2004). 15118073 10.1056/NEJMoa040938

[13] Zhou, C. C. et al. Amivantamab plus chemotherapy in NSCLC with EGFR exon 20 insertions. *N. Engl. J. Med.* **389**, 2039–2051 (2023). 37870976 10.1056/NEJMoa2306441

[14] Hanahan, D. & Weinberg, R. A. Hallmarks of cancer: the next generation. *Cell* **144**, 646–674 (2011). 21376230 10.1016/j.cell.2011.02.013

[15] Jing, X. et al. Role of hypoxia in cancer therapy by regulating the tumor microenvironment. *Mol. Cancer* **18**, 157 (2019). 31711497 10.1186/s12943-019-1089-9 PMC6844052

[16] Bhandari, V. et al. Molecular landmarks of tumor hypoxia across cancer types. *Nat. Genet.* **51**, 308–318 (2019). 30643250 10.1038/s41588-018-0318-2

[17] Mamo, M. et al. Hypoxia alters the response to anti-EGFR therapy by regulating EGFR expression and downstream signaling in a DNA methylation–specific and HIF-dependent manner. *Cancer Res.* **80**, 4998–5010 (2020). 33023947 10.1158/0008-5472.CAN-20-1232 PMC7669740

[18] Rego, R. L. et al. Prognostic effect of activated EGFR expression in human colon carcinomas: comparison with EGFR status. *Br. J. Cancer* **102**, 165–172 (2010). 19997103 10.1038/sj.bjc.6605473 PMC2813748

[19] Bhan, A., Soleimani, M. & Mandal, S. S. Long noncoding RNA and cancer: a new paradigm. *Cancer Res.* **77**, 3965–3981 (2017). 28701486 10.1158/0008-5472.CAN-16-2634 PMC8330958

[20] Schmitt, A. M. & Chang, H. Y. Long noncoding RNAs in cancer pathways. *Cancer Cell* **29**, 452–463 (2016). 27070700 10.1016/j.ccell.2016.03.010 PMC4831138

[21] Santini, J. et al. Characterization, quantification, and potential clinical-value of the epidermal growth-factor receptor in head and neck squamous-cell carcinomas. *Head Neck* **13**, 132–139 (1991). 2022478 10.1002/hed.2880130209

[22] Mok, T. S. et al. Gefitinib or carboplatin-paclitaxel in pulmonary adenocarcinoma. *N. Engl. J. Med.* **361**, 947–957 (2009). 19692680 10.1056/NEJMoa0810699

[23] Borgeaud, M. et al. Unveiling the landscape of uncommon EGFR mutations in NSCLC—a systematic review. *J. Thorac. Oncol.* **19**, 973–983 (2024). 38499147 10.1016/j.jtho.2024.03.016

[24] Sigismund, S. et al. Clathrin-mediated internalization is essential for sustained EGFR signaling but dispensable for degradation. *Dev. Cell* **15**, 209–219 (2008). 18694561 10.1016/j.devcel.2008.06.012

[25] Sigismund, S. et al. Threshold-controlled ubiquitination of the EGFR directs receptor fate. *EMBO J.* **32**, 2140–2157 (2013). 23799367 10.1038/emboj.2013.149 PMC3730230

[26] Bisson, N. et al. Selected reaction monitoring mass spectrometry reveals the dynamics of signaling through the GRB2 adaptor. *Nat. Biotechnol.* **29**, 653–658 (2011). 21706016 10.1038/nbt.1905

[27] Haglund, K. et al. Multiple monoubiquitination of RTKs is sufficient for their endocytosis and degradation. *Nat. Cell Biol.* **5**, 461–466 (2003). 12717448 10.1038/ncb983

[28] Schito, L. & Semenza, G. L. Hypoxia-inducible factors: master regulators of cancer progression. *Trends Cancer* **2**, 758–770 (2016). 28741521 10.1016/j.trecan.2016.10.016

[29] Uribe, M. L., Marrocco, I. & Yarden, Y. EGFR in cancer: signaling mechanisms, drugs, and acquired resistance. *Cancers* **13**, 2748 (2021). 34206026 10.3390/cancers13112748 PMC8197917

[30] Schmitt, N. C., Trivedi, S. & Ferris, R. L. STAT1 activation is enhanced by cisplatin and variably affected by EGFR inhibition in HNSCC cells. *Mol. Cancer Ther.* **14**, 2103–2111 (2015). 26141950 10.1158/1535-7163.MCT-15-0305 PMC4561003

[31] Banerjee, K. & Resat, H. Constitutive activation of STAT3 in breast cancer cells: a review. *Int. J. Cancer* **138**, 2570–2578 (2016). 26559373 10.1002/ijc.29923 PMC4801660

[32] Xue, H. et al. A novel tumor-promoting mechanism of IL6 and the therapeutic efficacy of tocilizumab: hypoxia-induced IL6 is a potent autophagy initiator in glioblastoma via the p-STAT3–MIR155–3p-CREBRF pathway. *Autophagy* **12**, 1129–1152 (2016). 27163161 10.1080/15548627.2016.1178446 PMC4990999

[33] Meng, J. et al. ID1 confers cancer cell chemoresistance through STAT3/ATF6-mediated induction of autophagy. *Cell Death Dis* **11**, 137 (2020). 32080166 10.1038/s41419-020-2327-1 PMC7033197

[34] Zhang, J. & Ney, P. A. Role of BNIP3 and NIX in cell death, autophagy, and mitophagy. *Cell Death Differ.* **16**, 939–946 (2009). 19229244 10.1038/cdd.2009.16 PMC2768230

[35] Milani, M. et al. The role of ATF4 stabilization and autophagy in resistance of breast cancer cells treated with bortezomib. *Cancer Res.* **69**, 4415–4423 (2009). 19417138 10.1158/0008-5472.CAN-08-2839

[36] Lee, J. G. et al. Autophagy contributes to the chemo-resistance of non-small cell lung cancer in hypoxic conditions. *Respir. Res* **16**, 138 (2015). 26553068 10.1186/s12931-015-0285-4 PMC4640373

[37] Nukaga, S. et al. Amplification of EGFR wild-type alleles in non-small cell lung cancer cells confers acquired resistance to mutation-selective EGFR tyrosine kinase inhibitors. *Cancer Res.* **77**, 2078–2089 (2017). 28202511 10.1158/0008-5472.CAN-16-2359

[38] Maycotte, P. et al. STAT3-mediated autophagy dependence identifies subtypes of breast cancer where autophagy inhibition can be efficacious. *Cancer Res.* **74**, 2579–2590 (2014). 24590058 10.1158/0008-5472.CAN-13-3470 PMC4008672

[39] Ono, M. & Kuwano, M. Molecular mechanisms of epidermal growth factor receptor (EGFR) activation and response to gefitinib and other EGFR-targeting drugs. *Clin. Cancer Res.* **12**, 7242–7251 (2006). 17189395 10.1158/1078-0432.CCR-06-0646

[40] Guo, G. et al. Ligand-independent EGFR signaling. *Cancer Res.* **75**, 3436–3441 (2015). 26282175 10.1158/0008-5472.CAN-15-0989 PMC4558210

[41] Zhang, X., Gureasko, J., Shen, K., Cole, P. A. & Kuriyan, J. An allosteric mechanism for activation of the kinase domain of epidermal growth factor receptor. *Cell.* **125**, 1137–1149 (2006). 16777603 10.1016/j.cell.2006.05.013

[42] Liu, H., Zhang, B. & Sun, Z. Spectrum of EGFR aberrations and potential clinical implications: insights from integrative pan-cancer analysis. *Cancer Commun.* **40**, 43–59 (2020). 10.1002/cac2.12005 PMC7163653 32067422

[43] Rehmani, H. S. & Issaeva, N. EGFR in head and neck squamous cell carcinoma: exploring possibilities of novel drug combinations. *Ann. Transl. Med* **8**, 813 (2020). 32793658 10.21037/atm.2020.04.07 PMC7396252

[44] Kenny, P. A. & Bissell, M. J. Targeting TACE-dependent EGFR ligand shedding in breast cancer. *J. Clin. Investig.* **117**, 337–345 (2007). 17218988 10.1172/JCI29518 PMC1764856

[45] Sutton, P., Borgia, J. A. & Bonomi, P. & Plate, J. M. D. Lyn, a Src family kinase, regulates activation of epidermal growth factor receptors in lung adenocarcinoma cells. *Mol. Cancer* **12**, 76 (2013). 23866081 10.1186/1476-4598-12-76 PMC3725175

[46] de Heer, E. C., Jalving, M. & Harris, A. L. HIFs, angiogenesis, and metabolism: elusive enemies in breast cancer. *J. Clin. Investig.* **130**, 5074–5087 (2020). 32870818 10.1172/JCI137552 PMC7524491

[47] Franovic, A. et al. Translational up-regulation of the EGFR by tumor hypoxia provides a nonmutational explanation for its overexpression in human cancer. *Proc. Natl Acad. Sci. USA* **104**, 13092–13097 (2007). 17670948 10.1073/pnas.0702387104 PMC1941796

[48] Wang, Y. et al. Hypoxia promotes ligand-independent EGF receptor signaling via hypoxia-inducible factor-mediated upregulation of caveolin-1. *Proc. Natl Acad. Sci. USA* **109**, 4892–4897 (2012). 22411794 10.1073/pnas.1112129109 PMC3323978

[49] Pascolutti, R. et al. Molecularly distinct clathrin-coated pits differentially impact EGFR fate and signaling. *Cell Rep.* **27**, 3049–3061.e3046 (2019). 31167147 10.1016/j.celrep.2019.05.017 PMC6581797

[50] Kroemer, G., Mariño, G. & Levine, B. Autophagy and the integrated stress response. *Mol. Cell* **40**, 280–293 (2010). 20965422 10.1016/j.molcel.2010.09.023 PMC3127250

[51] Wu, M. & Zhang, P. EGFR-mediated autophagy in tumourigenesis and therapeutic resistance. *Cancer Lett.* **469**, 207–216 (2020). 31639425 10.1016/j.canlet.2019.10.030

[52] Bao, X. et al. Proteolytic release of the p75(NTR) intracellular domain by ADAM10 promotes metastasis and resistance to anoikis. *Cancer Res.* **78**, 2262–2276 (2018). 29437707 10.1158/0008-5472.CAN-17-2789

[53] Liao, H. W. et al. PRMT1-mediated methylation of the EGF receptor regulates signaling and cetuximab response. *J. Clin. Investig.* **125**, 4529–4543 (2015). 26571401 10.1172/JCI82826 PMC4665782

[54] Chakraborty, A. et al. Casein kinase-2 mediates cell survival through phosphorylation and degradation of inositol hexakisphosphate kinase-2. *Proc. Natl Acad. Sci. USA* **108**, 2205–2209 (2011). 21262846 10.1073/pnas.1019381108 PMC3038742

[55] Lonic, A. et al. Phosphorylation of PKC delta by FER tips the balance from EGFR degradation to recycling. *J. Cell Biol.* **220**, e201902073 (2021). 33411917 10.1083/jcb.201902073 PMC7797899

[56] Zhang, X. W. et al. Neurokinin-1 receptor promotes non-small cell lung cancer progression through transactivation of EGFR. *Cell Death Disease* **13**, 41 (2022). 35013118 10.1038/s41419-021-04485-y PMC8748918

[57] Alwan, H. A. J., van Zoelen, E. J. J. & van Leeuwen, J. E. M. Ligand-induced lysosomal epidermal growth factor receptor (EGFR) degradation is preceded by proteasome-dependent EGFR de-ubiquitination. *J. Biol. Chem.* **278**, 35781–35790 (2003). 12829707 10.1074/jbc.M301326200

[58] Shi, J. B., Liu, Z. Y. & Xu, Q. Tumor necrosis factor receptor-associated factor 6 contributes to malignant behavior of human cancers through promoting AKT ubiquitination and phosphorylation. *Cancer Sci.* **110**, 1909–1920 (2019). 30945383 10.1111/cas.14012 PMC6549921

[59] Xu, Q., Liu, X., Chen, W. & Zhang, Z. Inhibiting adenoid cystic carcinoma cells growth and metastasis by blocking the expression of ADAM 10 using RNA interference. *J. Transl. Med.* **8**, 136 (2010). 21171968 10.1186/1479-5876-8-136 PMC3017514

