# Supplementary material for: LncRNA EUDAL shapes tumor cell response to hypoxia-induced constitutive EGFR activation and promotes chemoresistance in oral cancer
Source: Int J Oral Sci. 2025 Sep 12;17:64. doi: 10.1038/s41368-025-00396-2 (PMC12432167; doi:10.1038/s41368-025-00396-2)
Supplement: Supplementary file 1 — Supplementary Material [file 41368_2025_396_MOESM1_ESM.pdf]

Supplementary Material for

LncRNA EUDAL shapes tumor cell response to hypoxia-induced constitutive EGFR activation and promotes chemoresistance in oral cancer

List of supplementary information

Supplementary methods.

Supplementary Figures S1 to S12 for multiple supplementary data figures.

Supplementary Tables S1 to S4 for multiple supplementary data tables.

SUPPLEMENTARY METHODS

Colony formation assay

Approximately 1,000 cells were planted in the cultivation well (Φ 3 cm). After 2 weeks of culture under normoxic or hypoxic conditions, the colonies were fixed with 4% paraformaldehyde, and the cells were stained with crystal violet solution.

Cell motility assay

Cell motility was assessed via a scratch test. After the cells reached 100% confluence, a scratch was made in the middle of each culture well. Microscopy images were taken immediately after 12 h and after 24 h under hypoxic or normoxic conditions.

Cell apoptosis assay

The percentage of cells undergoing apoptosis after different treatments was determined via Annexin V-propidium iodide (PI) staining followed by flow cytometry. Annexin V-positive cells were considered apoptotic cells.

EGFR exon sequencing

Primers specific to exons 18-21 was designed and the PCR product was isolated using DNA purification kit (Sangong Biotech, Shanghai, China). Sanger sequencing was implemented using 3730xl DNA Analyzer (Applied Biosystems, USA). Sequencing results were aligned with NCBI reference sequence NG_007726.3. Primers used were listed in Supplementary Table S1.

Figure S1:

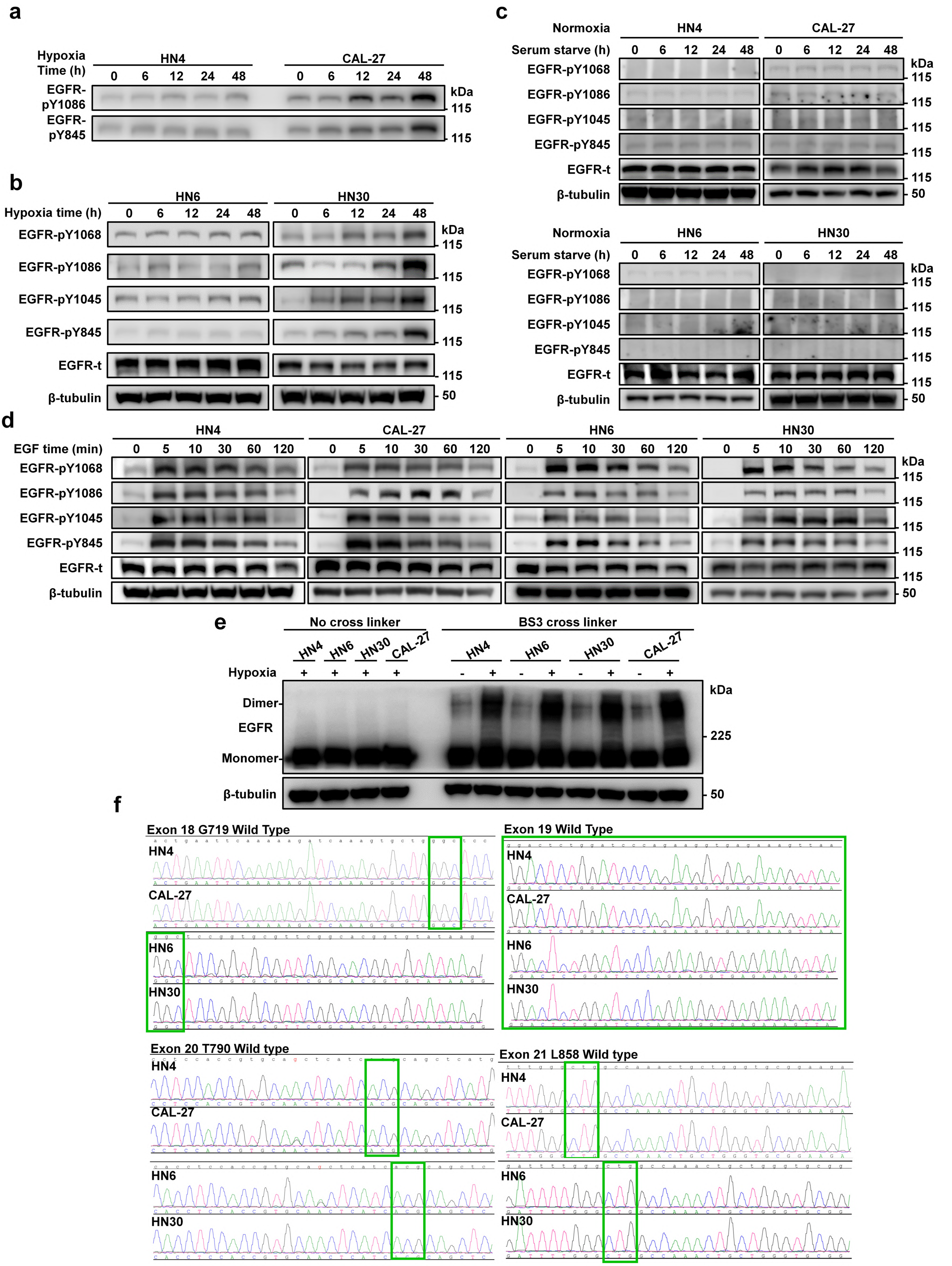
a
Hypoxia Time (h)
HN4
CAL-27
0 6 12 24 48
EGFR-pY1086
EGFR-pY845
kDa
115
b
Hypoxia time (h)
HN6
HN30
EGFR-pY1068
EGFR-pY1086
EGFR-pY1045
EGFR-pY845
EGFR-t
β-tubulin
50
c
Normoxia
Serum starve (h)
d
EGF time (min)
0 5 10 30 60 120
e
No cross linker
BS3 cross linker
Hypoxia
Dimer
EGFR
Monomer
225
f
Exon 18 G719 Wild Type
Exon 19 Wild Type
Exon 20 T790 Wild type
Exon 21 L858 Wild type

Fig. S1 EGFR phosphorylation in hypoxic or normoxic oral cancer cell lines harboring wild-type EGFR. **a-b** Hypoxia induced chronic but constitutive EGFR phosphorylation on a series of tyrosine residues. **c** pEGFR level of HN4, CAL-27, HN6, and HN30 under serum-free normoxic culture. **d** 20 ng/mL EGF-stimulated rapid but transient phosphorylation of EGFR of HN4, CAL-27, HN6, and HN30 cell lines. **e** Dimerization assay of oral cancer cells with or without hypoxia treatment. **f** Exon sequencing showing no activating mutation on endogenously expressed EGFR of HN4, CAL-27, HN6, and HN30 cell lines. Data were from representative results of at least three independent experiments.

Figure S2:

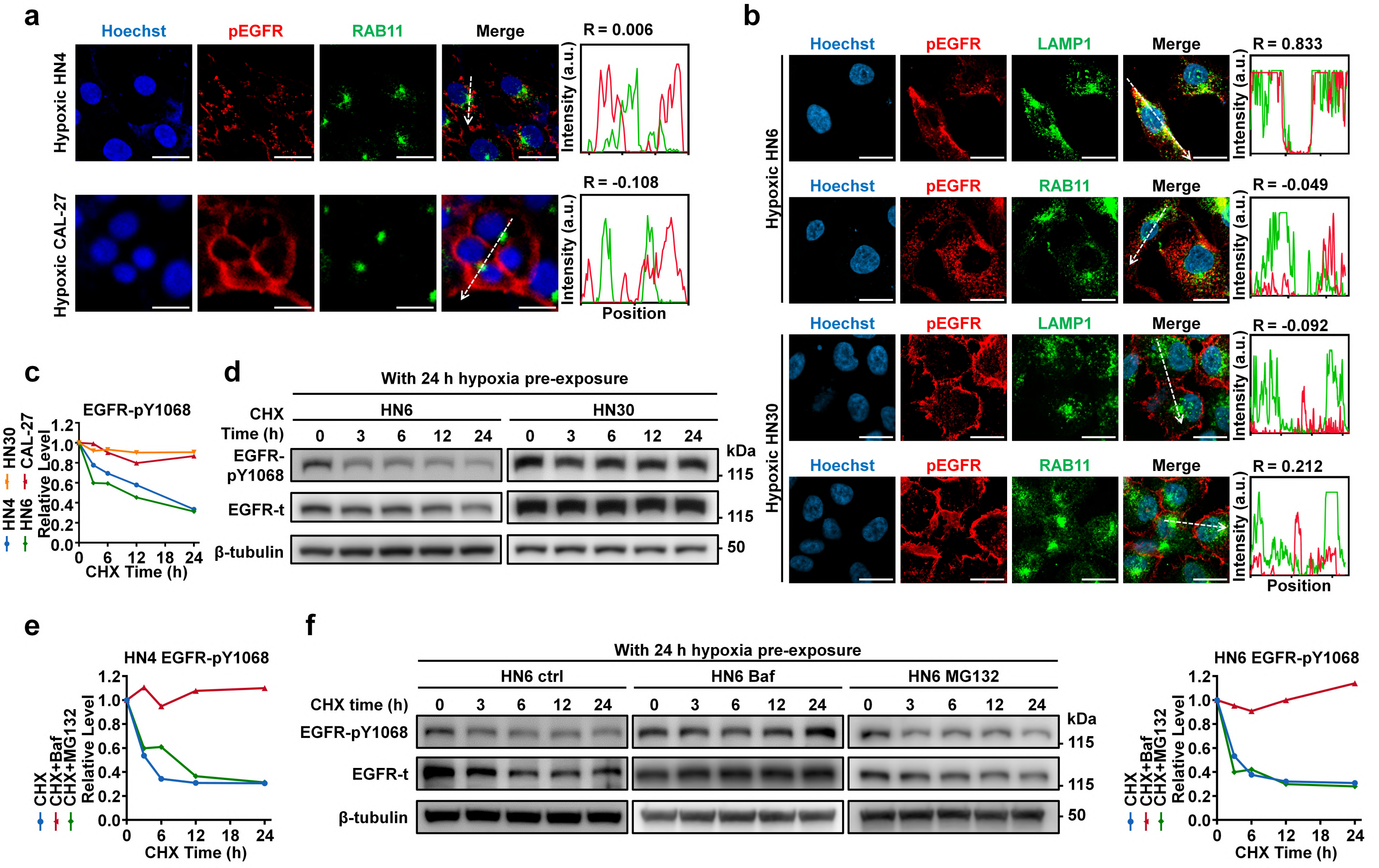

a
Hoechst
pEGFR
RAB11
Merge
Hypoxic HN4
Hypoxic CAL-27
R = 0.006
R = -0.108
Intensity (a.u.)
Position
b
Hoechst
pEGFR
LAMP1
RAB11
Merge
Hypoxic HN6
Hypoxic HN30
R = 0.833
R = -0.049
R = -0.092
R = 0.212
Intensity (a.u.)
Position
c
EGFR-pY1068
HN4
HN6
HN30
CAL-27
Relative Level
CHX Time (h)
d
With 24 h hypoxia pre-exposure
CHX Time (h)
HN6
HN30
0 3 6 12 24
EGFR-pY1068
EGFR-t
β-tubulin
kDa
115
115
50
e
HN4 EGFR-pY1068
CHX
CHX+Baf
CHX+MG132
Relative Level
CHX Time (h)
f
With 24 h hypoxia pre-exposure
HN6 ctrl
HN6 Baf
HN6 MG132
CHX time (h)
0 3 6 12 24
EGFR-pY1068
EGFR-t
β-tubulin
kDa
115
115
50
HN6 EGFR-pY1068
CHX
CHX+Baf
CHX+MG132
Relative Level
CHX Time (h)

Fig. S2 Endocytosis, subcellular localization, and lysosomal degradation of EGFR in oral cancer cells. **a-b** The colocalization of pEGFR with the lysosome marker LAMP1 and the recycling pathway marker RAB11 in hypoxic HN4, CAL-27, HN6, and HN30 cells as shown by confocal microscopy images. The colocalization of pEGFR (red) and LAMP1/RAB11 (green) was estimated with plot profile using ImageJ. Correlation coefficient $R > 0.6$ was considered well-collocated while $R < 0.6$ was taken as poorly-collocated. Bar, 20 μm. **c** Densitometric analysis of WB results in Fig. 1e and Supplementary Fig. S1d. **d** Representative immunoblot images and half-life analysis of pEGFR and total EGFR levels of HN6 and HN30 cells after CHX treatment for the indicated time periods. **e** Densitometric analysis of WB results in Fig. 2a. **f** Representative immunoblot images and half-life analysis of pEGFR and total EGFR levels of HN6 cells after CHX treatment in the absence and presence of 40 nM Bafilomycin A1 (Baf) or 5 μM MG132. Data were from representative results of at least three independent experiments. Data are represented as mean ± SD. ****, $p < 0.0001$; one-way ANOVA (**d**); Pearson's R correlation(**e**).

Figure S3:

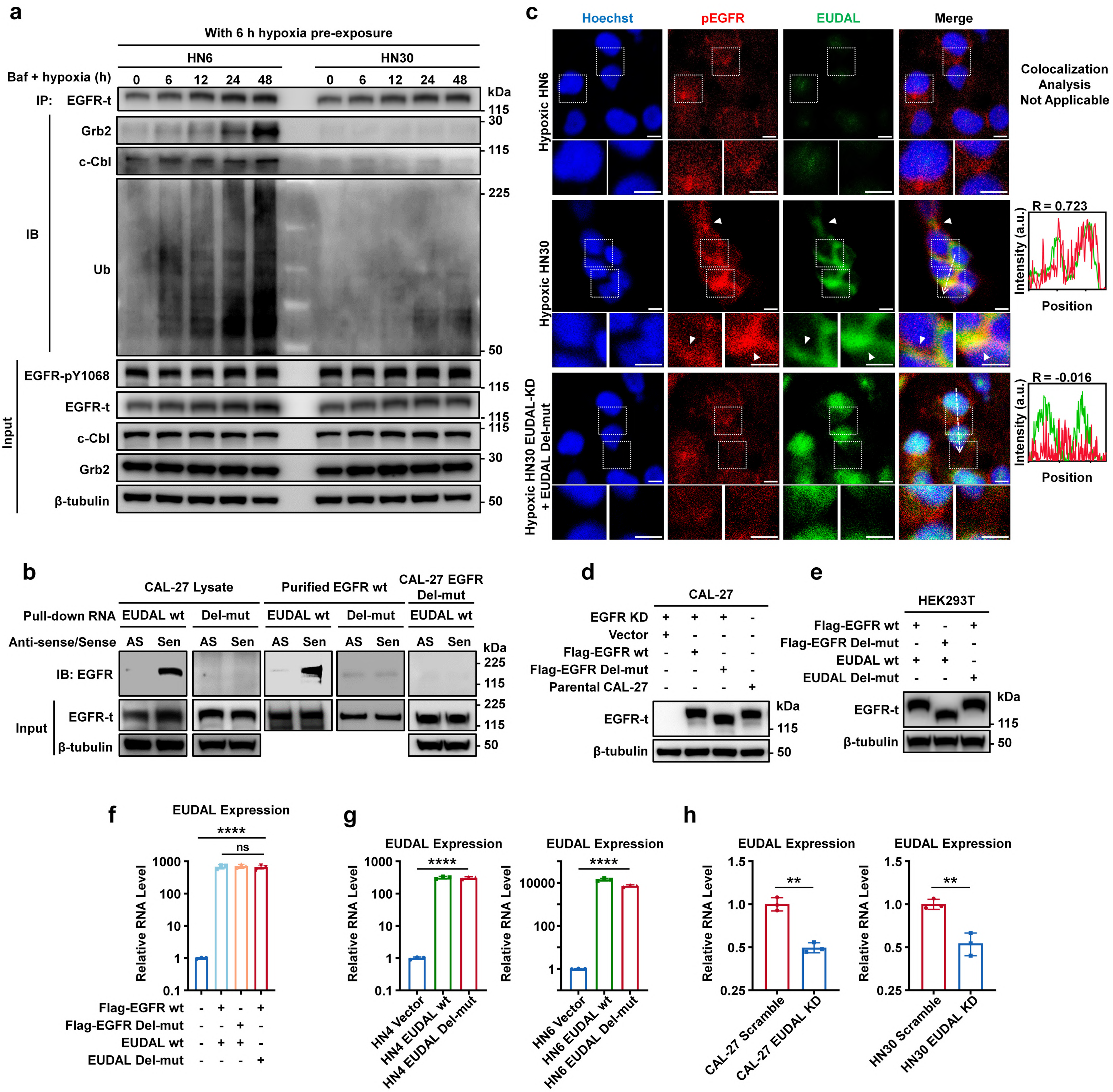
a
With 6 h hypoxia pre-exposure
HN6
HN30
Baf + hypoxia (h)
0 6 12 24 48
kDa
IP: EGFR-t
IB
Grb2
c-Cbl
Ub
Input
EGFR-pY1068
EGFR-t
β-tubulin
115
30
225
50
c
Hoechst
pEGFR
EUDAL
Merge
Hypoxic HN6
Hypoxic HN30
Hypoxic HN30 EUDAL-KD + EUDAL Del-mut
Colocalization Analysis Not Applicable
R = 0.723
R = -0.016
Intensity (a.u.)
Position
b
CAL-27 Lysate
Purified EGFR wt
CAL-27 EGFR Del-mut
Pull-down RNA
EUDAL wt
Del-mut
Anti-sense/Sense
AS
Sen
IB: EGFR
Input
EGFR-t
β-tubulin
d
CAL-27
EGFR KD
Vector
Flag-EGFR wt
Flag-EGFR Del-mut
Parental CAL-27
e
HEK293T
EUDAL wt
EUDAL Del-mut
f
EUDAL Expression
****
ns
Relative RNA Level
1000
100
10
1
0.1
g
HN4 Vector
HN4 EUDAL wt
HN4 EUDAL Del-mut
10000
HN6 Vector
HN6 EUDAL wt
HN6 EUDAL Del-mut
h
**
1.5
1.0
0.5
0.25
CAL-27 Scramble
CAL-27 EUDAL KD
HN30 Scramble
HN30 EUDAL KD

Fig. S3 EGFR degradation, binding with EUDAL, and validation of overexpression or knockdown involved. **a** A time-course analysis of EGFR ubiquitination and the interaction between EGFR and c-Cbl as well as Grb2 in hypoxic HN6 and HN30 cells. **b** RNA pull-down assays indicated a direct interaction between EUDAL and EGFR. **c** The colocalization of pEGFR and EUDAL in hypoxic HN6 and HN30 cells following indicated treatments as shown by confocal microscopy images. Representative colocalization areas were pointed with arrow heads. The colocalization of pEGFR (red) and EUDAL (green) was estimated with plot profile using ImageJ. Correlation coefficient R > 0.6 was considered well-collocated while R < 0.6 was taken as poorly-collocated. Bar, 10 μm. **d** Western blotting results of EGFR KD and forced expression of EGFR wt and EGFR Del-mut proteins in CAL-27 cells. **e** Western blotting results of expression of EGFR wt and EGFR Del-mut proteins in HEK293T cells. **f** Overexpression efficiency of EUDAL wt and EUDAL Del-mut in HEK293T cells (n = 3). **g** Overexpression efficiency of EUDAL wt and EUDAL Del-mut in HN4 and HN6 cells (n = 3). **h** Knockdown efficiency of EUDAL in CAL-27 and HN30 cells (n = 3). Data were from representative results of at least three independent experiments. Data are represented as mean ± SD. ns, no significance; **, $p < 0.01$, ****, $p < 0.0001$; one-way ANOVA with Turkey's Honestly Significant Difference test (**f** and **g**); unpaired Student's *t*-test (**h**). Baf, Bafilomycin A1; AS, antisense EUDAL; Sen, sense EUDAL; wt, wild-type; EUDAL Del-mut, a lncRNA mutant with an EGFR-binding motif deletion (Δ254-305 nt); KD, knockdown.

Figure S4:

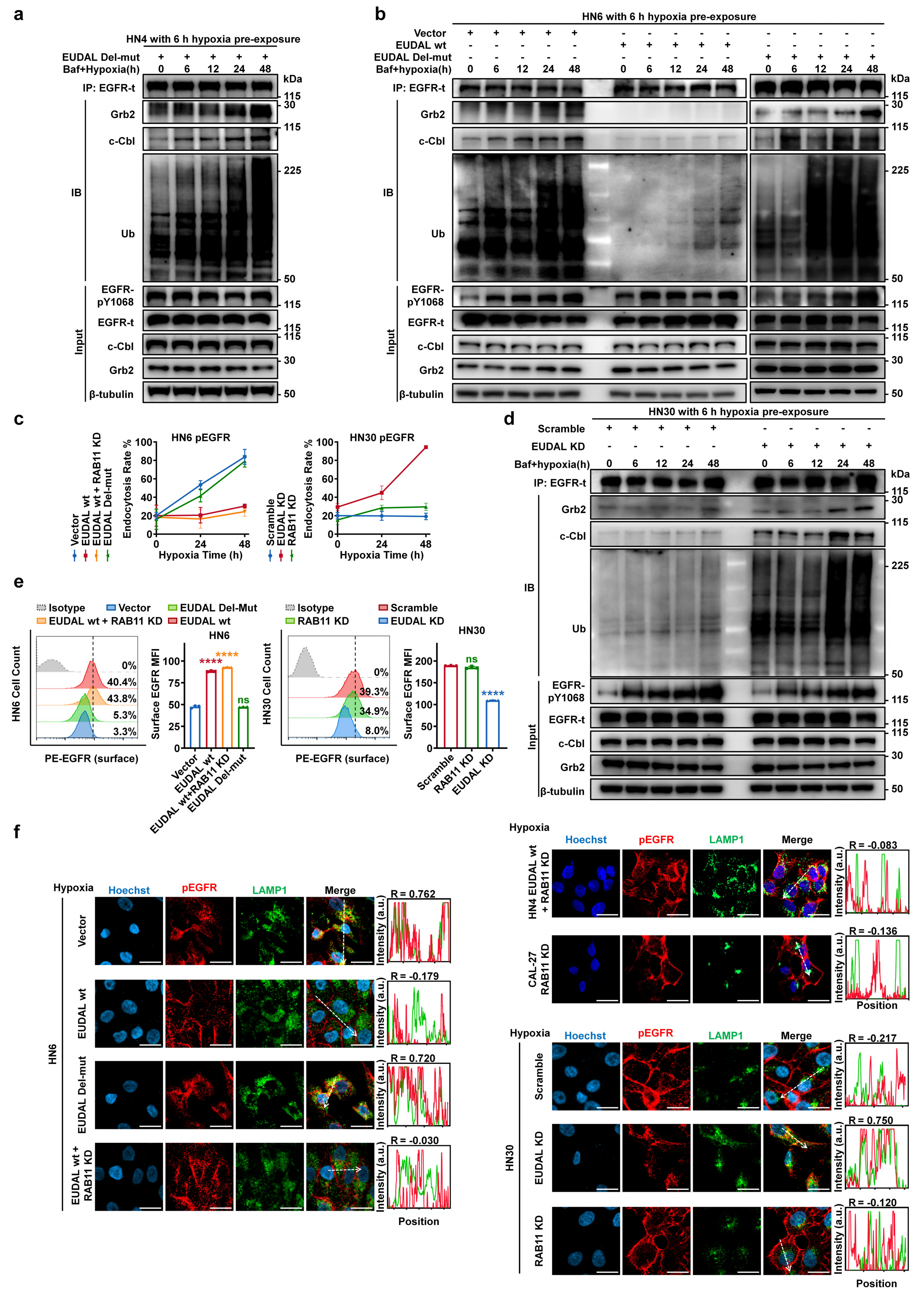

a
HN4 with 6 h hypoxia pre-exposure
EUDAL Del-mut
Baf+Hypoxia(h)
IP: EGFR-t
IB
Grb2
c-Cbl
Ub
Input
EGFR-pY1068
EGFR-t
c-Cbl
Grb2
β-tubulin
kDa
b
HN6 with 6 h hypoxia pre-exposure
Vector
EUDAL wt
EUDAL Del-mut
Baf+hypoxia(h)
c
HN6 pEGFR
HN30 pEGFR
Endocytosis Rate %
Hypoxia Time (h)
Vector
EUDAL wt
EUDAL wt + RAB11 KD
EUDAL Del-mut
Scramble
EUDAL KD
RAB11 KD
d
HN30 with 6 h hypoxia pre-exposure
Scramble
EUDAL KD
Baf+hypoxia(h)
e
Isotype
Vector
EUDAL Del-Mut
EUDAL wt + RAB11 KD
EUDAL wt
Scramble
RAB11 KD
EUDAL KD
HN6 Cell Count
HN30 Cell Count
PE-EGFR (surface)
Surface EGFR MFI
f
Hypoxia
Hoechst
pEGFR
LAMP1
Merge
HN6
HN30
HN4 EUDAL wt + RAB11 KD
CAL-27 RAB11 KD
Intensity (a.u.)
Position

Fig. S4 Ubiquitination, endocytosis, and lysosomal degradation of EGFR in oral cancer cells. **a** The effects of ectopic expression of EUDAL Del-mut on the interaction between c-Cbl/Grb2 and EGFR, and subsequent ubiquitination in hypoxic HN4 cells. **b** The ectopic expression of EUDAL wt blocked the interaction between c-Cbl/Grb2 and EGFR, and attenuated subsequent ubiquitination in hypoxic HN6 cells. **c** pEGFR endocytosis rate in hypoxic HN6 and HN30 following indicated treatments. **d** Silencing of EUDAL promoted binding of c-Cbl/Grb2 to EGFR and elevated EGFR ubiquitination levels in hypoxic HN30 cells. **e** Flow cytometric analysis of membrane-localized EGFR on hypoxic HN6 and HN30 cells (n = 3). **f** The colocalization of pEGFR with the lysosome marker LAMP1 in hypoxic HN4, CAL-27, HN6, and HN30 cells following indicated treatments as shown by confocal microscopy images. The colocalization of pEGFR (red) and LAMP1 (green) was estimated with plot profile using ImageJ. Correlation coefficient R > 0.6 was considered well-collocated while R < 0.6 was taken as poorly-collocated. Bar, 20 μm. Pearson's R correlation (**f**). wt, wild-type; EUDAL Del-mut, a lncRNA mutant with an EGFR-binding motif deletion (Δ254-305 nt); KD, knockdown.

Figure S5:

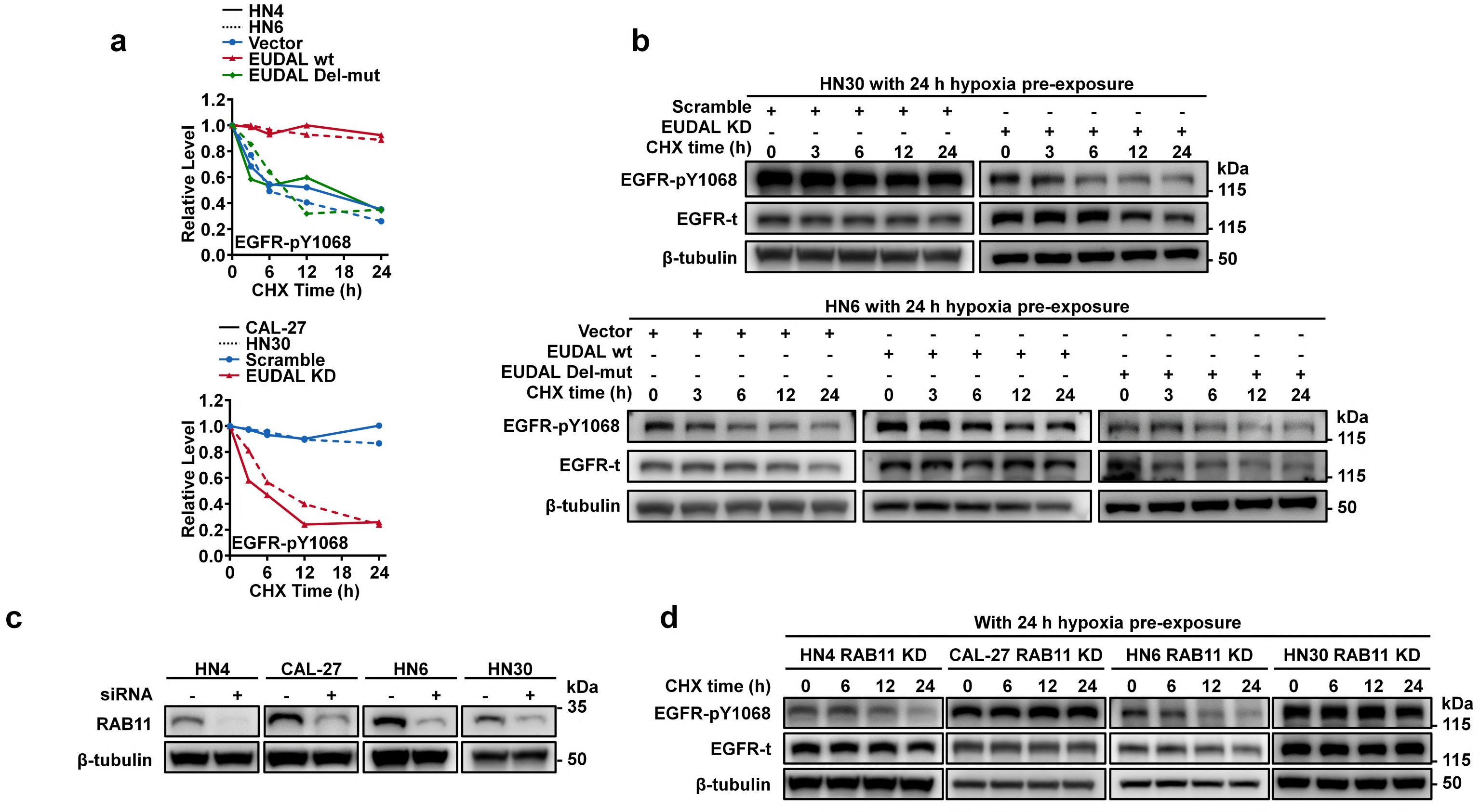

a
HN4
HN6
Vector
EUDAL wt
EUDAL Del-mut
Relative Level
EGFR-pY1068
CHX Time (h)
CAL-27
HN30
Scramble
EUDAL KD
b
HN30 with 24 h hypoxia pre-exposure
Scramble
EUDAL KD
CHX time (h)
EGFR-pY1068
EGFR-t
β-tubulin
kDa
115
50
HN6 with 24 h hypoxia pre-exposure
Vector
EUDAL wt
EUDAL Del-mut
c
HN4
CAL-27
HN6
HN30
siRNA
RAB11
35
d
With 24 h hypoxia pre-exposure
HN4 RAB11 KD
CAL-27 RAB11 KD
HN6 RAB11 KD
HN30 RAB11 KD

Fig. S5 Stability analysis of pEGFR after altering EUDAL or RAB11 expression. **a** pEGFR half-life analysis of results in Fig. 5b and Supplementary Fig. S5b. **b** Representative immunoblot images of pEGFR and total EGFR levels after CHX treatment following indicated treatments. **c** Western blotting results of RAB11 KD validation. **d** pEGFR levels after CHX (cycloheximide) treatment of RAB11 KD cells. Data were from representative results of at least three independent experiments. wt, wild-type; EUDAL Del-mut, a lncRNA mutant with an EGFR-binding motif deletion (Δ254-305 nt); KD, knockdown.

Figure S6:

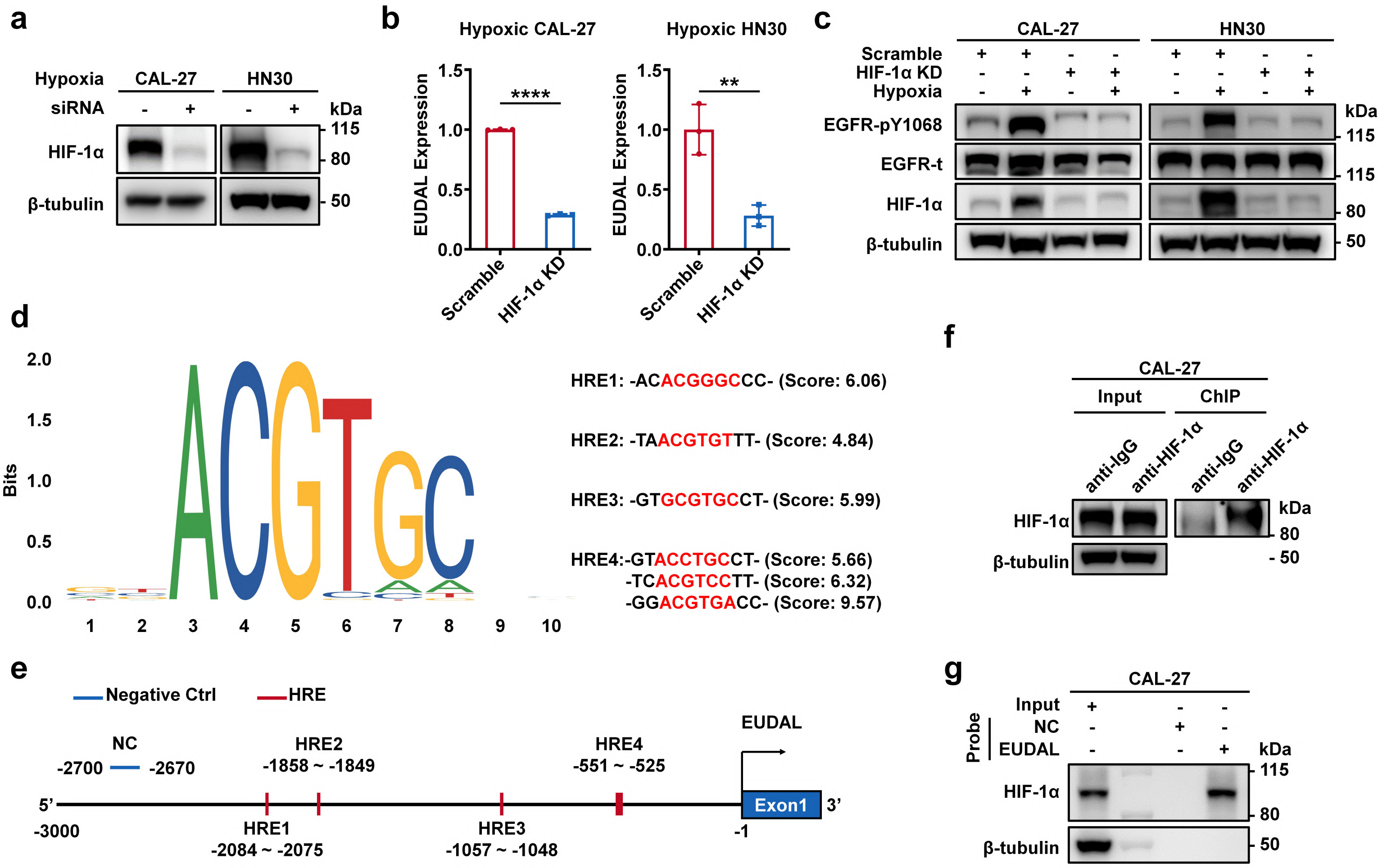
a
Hypoxia
CAL-27
HN30
siRNA
kDa
HIF-1α
β-tubulin
115
80
50
b
Hypoxic CAL-27
Hypoxic HN30
EUDAL Expression
****
**
Scramble
HIF-1α KD
c
CAL-27
HN30
Scramble
HIF-1α KD
Hypoxia
EGFR-pY1068
EGFR-t
HIF-1α
β-tubulin
kDa
115
115
80
50
d
Bits
HRE1: -ACACGGGCCC- (Score: 6.06)
HRE2: -TAACGTGTTT- (Score: 4.84)
HRE3: -GTGCGTGCCT- (Score: 5.99)
HRE4:-GTACCTGCCT- (Score: 5.66)
-TCACGTCCTT- (Score: 6.32)
-GGACGTGACC- (Score: 9.57)
e
Negative Ctrl
HRE
NC
-2700
-2670
HRE2
-1858 ~ -1849
HRE4
-551 ~ -525
EUDAL
5'
3'
Exon1
-3000
HRE1
-2084 ~ -2075
HRE3
-1057 ~ -1048
-1
f
CAL-27
Input
ChIP
anti-IgG
anti-HIF-1α
anti-IgG
anti-HIF-1α
HIF-1α
β-tubulin
kDa
80
50
g
CAL-27
Input
Probe
NC
EUDAL
HIF-1α
β-tubulin
kDa
115
80
50

Fig. S6 HIF-1α transcriptionally activates EUDAL expression. **a** Expression level of HIF-1α in CAL-27 and HN30 cells after RNA interference in hypoxic condition. **b** Knockdown of HIF-1α dramatically reduced the expression level of EUDAL in hypoxic CAL-27 and HN30 cells ($n = 3$). **c** Knockdown of HIF-1α attenuated hypoxia-induced EGFR activation in CAL-27 and HN30 cells. **d** JASPAR prediction results of HIF-1α binding sequences in 3,000 bp upstream of EUDAL exon 1. Core HRE sequences (red) of HRE1-4 along with the JASPAR Score were listed. HRE4 contains 3 core HIF-1α-binding motifs clustering from -525 to -551 bp. **e** Four putative hypoxia response elements (HREs) on the promoter of EUDAL were predicted *in silico*. PCR primers corresponding to the HREs (HRE1, 2, 3 and 4) and a non-HREs (NC) region of HIF-1α promoter were designed. **f** Cross-linked HIF-1α-chromatin complex was immunoprecipitated by anti-HIF1α antibody. **g** Reverse ChIP experiment showed that the promoter region of EUDAL could bind to HIF-1α. Data were from representative results of at least three independent experiments. **, $p < 0.01$; ****, $p < 0.0001$; unpaired Student's t-test (**b**); HIF-1α KD, knockdown of HIF-1α; NC, negative control.

Figure S7:

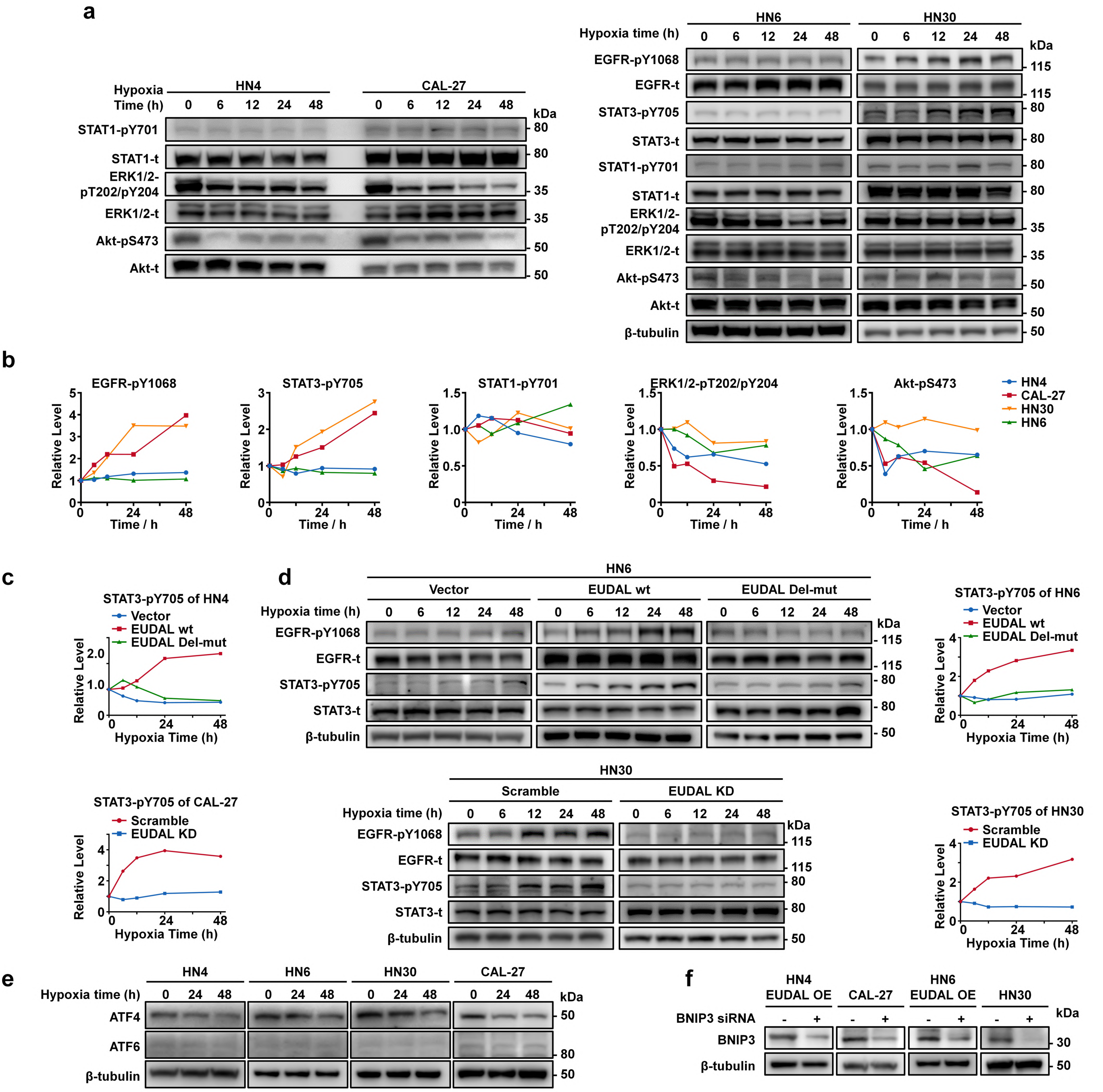
a
Hypoxia Time (h)
HN4
CAL-27
0 6 12 24 48
kDa
STAT1-pY701
STAT1-t
ERK1/2-pT202/pY204
ERK1/2-t
Akt-pS473
Akt-t
HN6
HN30
Hypoxia time (h)
EGFR-pY1068
EGFR-t
STAT3-pY705
STAT3-t
β-tubulin
b
EGFR-pY1068
STAT3-pY705
STAT1-pY701
ERK1/2-pT202/pY204
Akt-pS473
Relative Level
Time / h
HN4
CAL-27
HN30
HN6
c
STAT3-pY705 of HN4
Vector
EUDAL wt
EUDAL Del-mut
Hypoxia Time (h)
STAT3-pY705 of CAL-27
Scramble
EUDAL KD
d
STAT3-pY705 of HN6
STAT3-pY705 of HN30
e
ATF4
ATF6
f
HN4 EUDAL OE
HN6 EUDAL OE
BNIP3 siRNA
BNIP3

Fig. S7 Hypoxia mediated ligand-independent EGFR phosphorylation leads to activation of STAT3 signaling in oral cancer cells. **a** Representative immunoblot images of indicated protein levels. **b-c** Densitometric analysis of indicated protein levels in Fig. 6d and Supplementary Fig. S7a (**b**) and Fig. 6e (**c**). **d** Left Top: Hypoxia treatment combined with ectopic expression of EUDAL can simultaneously lead to phosphorylation of EGFR and STAT3 in HN6 cells. Left Bottom: Knockdown of EUDAL in HN30 cells inhibited phosphorylation of both EGFR and STAT3. Right: Densitometric analysis of STAT3-pY705 level. **e** Protein levels of ATF4 and ATF6 in HN4, HN6, HN30, and CAL-27 cells under hypoxic treatment. **f** Validation of BNIP3 knockdown. Data were from representative results of at least three independent experiments. wt, wild-type; EUDAL Del-mut, a lncRNA mutant with an EGFR-binding motif deletion (Δ254-305 nt); KD, knockdown.

Figure S8:

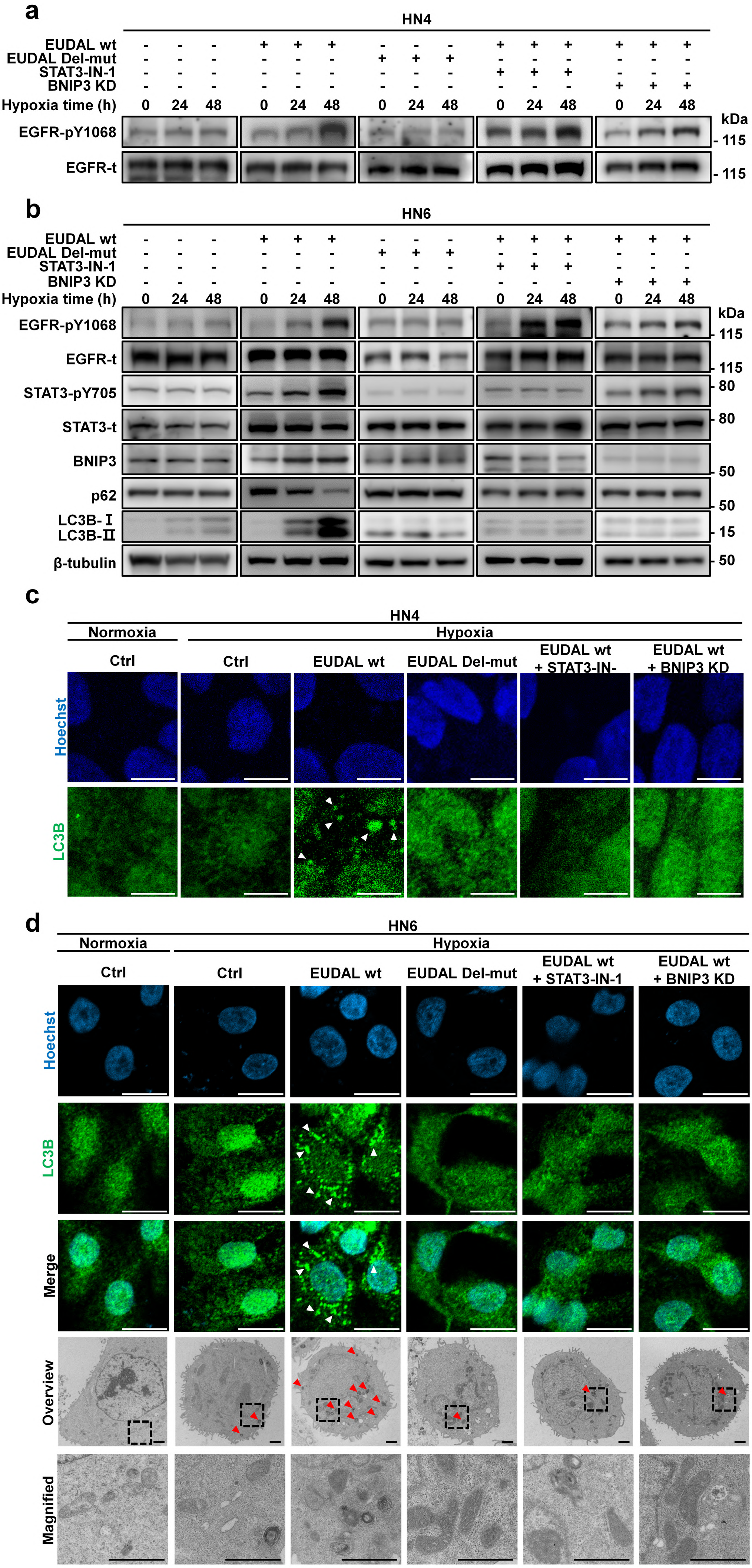
a
HN4
EUDAL wt
EUDAL Del-mut
STAT3-IN-1
BNIP3 KD
Hypoxia time (h)
0 24 48 0 24 48 0 24 48 0 24 48 0 24 48
EGFR-pY1068
EGFR-t
kDa
115
115
b
HN6
EUDAL wt
EUDAL Del-mut
STAT3-IN-1
BNIP3 KD
Hypoxia time (h)
EGFR-pY1068
EGFR-t
STAT3-pY705
STAT3-t
BNIP3
p62
LC3B-I
LC3B-II
β-tubulin
kDa
115
115
80
80
50
50
15
50
c
HN4
Normoxia
Hypoxia
Ctrl
Ctrl
EUDAL wt
EUDAL Del-mut
EUDAL wt + STAT3-IN-
EUDAL wt + BNIP3 KD
Hoechst
LC3B
d
HN6
Normoxia
Hypoxia
Ctrl
Ctrl
EUDAL wt
EUDAL Del-mut
EUDAL wt + STAT3-IN-1
EUDAL wt + BNIP3 KD
Hoechst
LC3B
Merge
Overview
Magnified

Fig. S8 Hypoxia/EUDAL/STAT3 axis activation results in autophagy in HN4 and HN6 cells. **a-b** Expression analysis of pEGFR in HN4 (**a**) and autophagy related markers LC3B and p62 as well as pEGFR, pSTAT3, and BNIP3 in HN6 cells following indicated treatments (**b**). **c** Individual channels of LC3B puncta (white arrowed) identified by fluorescence microscopy in HN4. **d** Upper: LC3B puncta (white arrowed) identified by fluorescence microscopy in HN6 cells following indicated treatments. Bar for fluorescent microscopies, 10 μm. Lower: Examination of autophagosome (red arrowed) in HN6 cells following indicated treatments by transmission electron microscopy. Bar for electronic microscopies, 1 μm. Data were from representative results of at least three independent experiments. wt, wild-type; EUDAL Del-mut, a lncRNA mutant with an EGFR-binding motif deletion (Δ254-305 nt); KD, knockdown.

Figure S9:

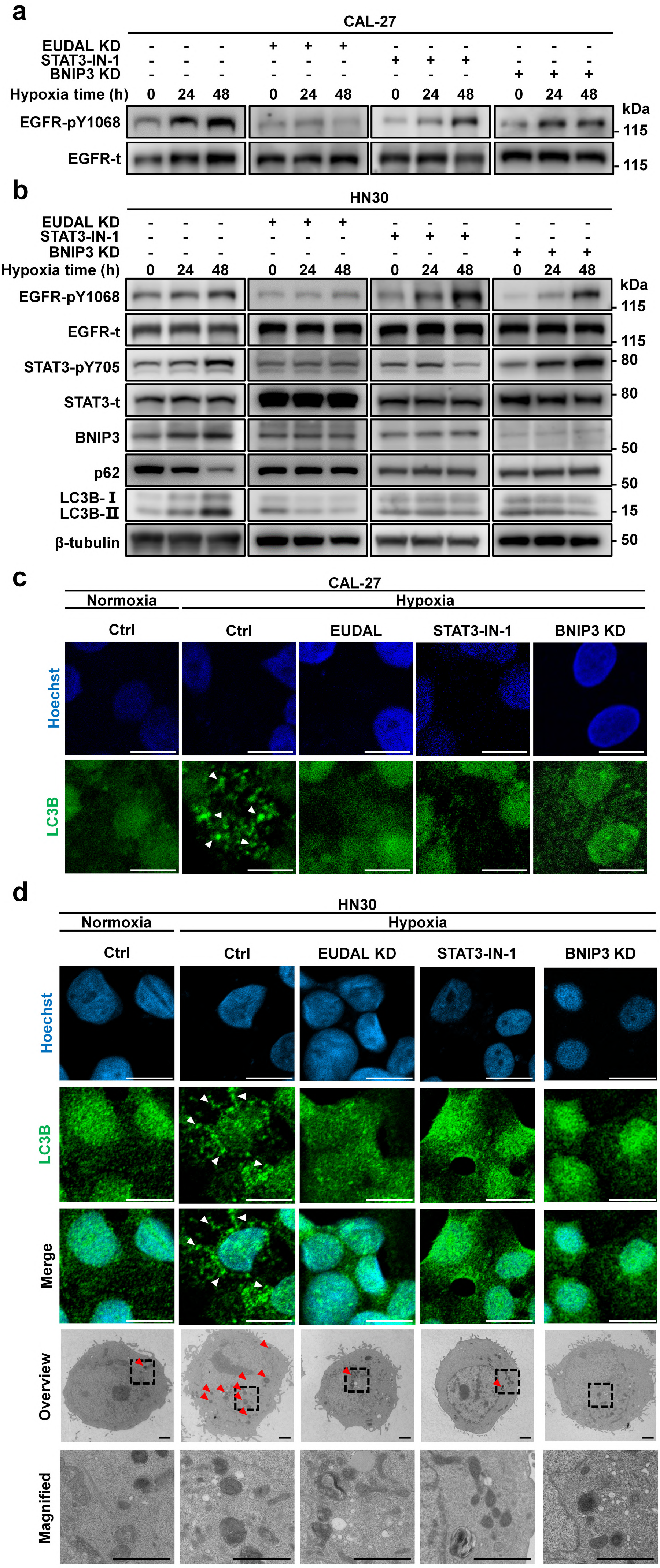
a
CAL-27
EUDAL KD
STAT3-IN-1
BNIP3 KD
Hypoxia time (h)
0 24 48 0 24 48 0 24 48 0 24 48
EGFR-pY1068
EGFR-t
kDa
115
115
b
HN30
EUDAL KD
STAT3-IN-1
BNIP3 KD
Hypoxia time (h)
EGFR-pY1068
EGFR-t
STAT3-pY705
STAT3-t
BNIP3
p62
LC3B-Ⅰ
LC3B-Ⅱ
β-tubulin
kDa
115
115
80
80
50
50
15
50
c
CAL-27
Normoxia
Hypoxia
Ctrl
Ctrl
EUDAL
STAT3-IN-1
BNIP3 KD
Hoechst
LC3B
d
HN30
Normoxia
Hypoxia
Ctrl
Ctrl
EUDAL KD
STAT3-IN-1
BNIP3 KD
Hoechst
LC3B
Merge
Overview
Magnified

Fig. S9 Hypoxia/EUDAL/STAT3 axis activation results in autophagy in CAL-27 and HN30 cells. **a-b** Expression analysis of pEGFR in CAL-27 (**a**) and autophagy related markers LC3B and p62 as well as pEGFR, pSTAT3, and BNIP3 in HN30 cells following indicated treatments (**b**). **c** Individual channels of LC3B puncta (white arrowed) identified by fluorescence microscopy in CAL-27. **d** Upper: LC3B puncta (white arrowed) identified by fluorescence microscopy in HN30 cells following indicated treatments. Bar for fluorescent microscopies, 10 μm. Lower: Examination of autophagosome (red arrowed) in HN30 cells following indicated treatments by transmission electron microscopy. Bar for electronic microscopies, 1 μm. Data were from representative results of at least three independent experiments. wt, wild-type; EUDAL Del-mut, a lncRNA mutant with an EGFR-binding motif deletion (Δ254-305 nt); KD, knockdown.

Figure S10:

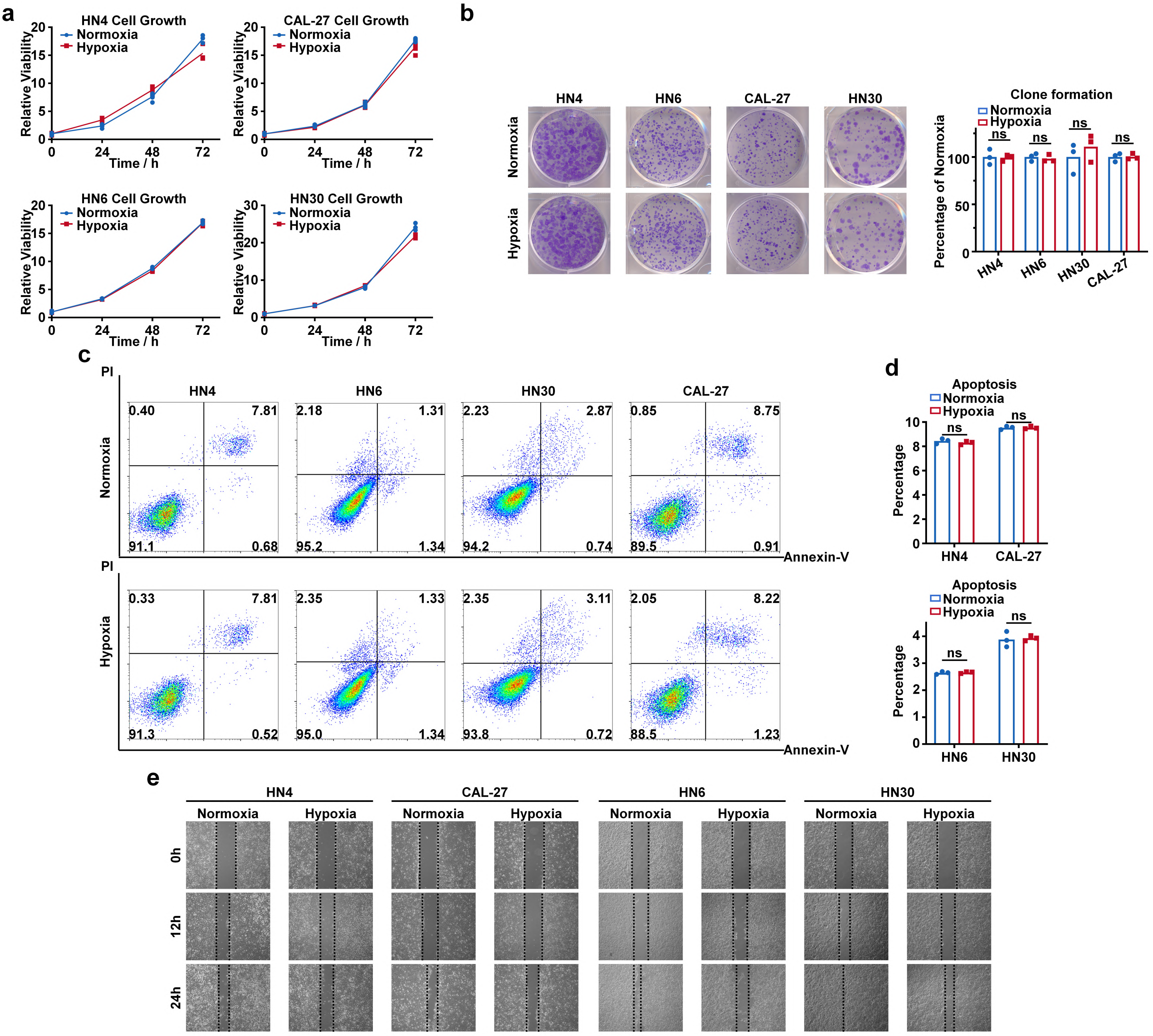
a
HN4 Cell Growth
Normoxia
Hypoxia
Relative Viability
Time / h
CAL-27 Cell Growth
HN6 Cell Growth
HN30 Cell Growth
b
HN4
HN6
CAL-27
HN30
Normoxia
Hypoxia
Clone formation
Percentage of Normoxia
ns
c
PI
Annexin-V
0.40 7.81 91.1 0.68
2.18 1.31 95.2 1.34
2.23 2.87 94.2 0.74
0.85 8.75 89.5 0.91
0.33 7.81 91.3 0.52
2.35 1.33 95.0 1.34
2.35 3.11 93.8 0.72
2.05 8.22 88.5 1.23
d
Apoptosis
Percentage
e
0h
12h
24h

Fig. S10 Biological behavior of normoxic or hypoxic oral cancer cell lines. **a** Cell proliferation rate of HN4, CAL-27, HN6, and HN30 showed no significant difference between normoxic and hypoxic cultivation (n = 5). **b** Clone formation assay of HN4, CAL-27, HN6, and HN30 under normoxic or hypoxic condition showed similar results (n = 3). **c-d** Similar apoptotic levels of all HN4, CAL-27, HN6, and HN30 cells under normoxic and hypoxic conditions were detected (n = 3). Representative flow cytometry results (**c**). Early and late apoptosis cells were counted as apoptotic cells (**d**). **e** Migration rate was slightly slower in hypoxic treatment but there was no significant difference between HN4, CAL-27, HN6, and HN30 (n = 3). Data were from representative results of at least three independent experiments. Data are represented as mean ± SD. ns, no significance; unpaired Student's t-test (**b** and **d**).

Figure S11:

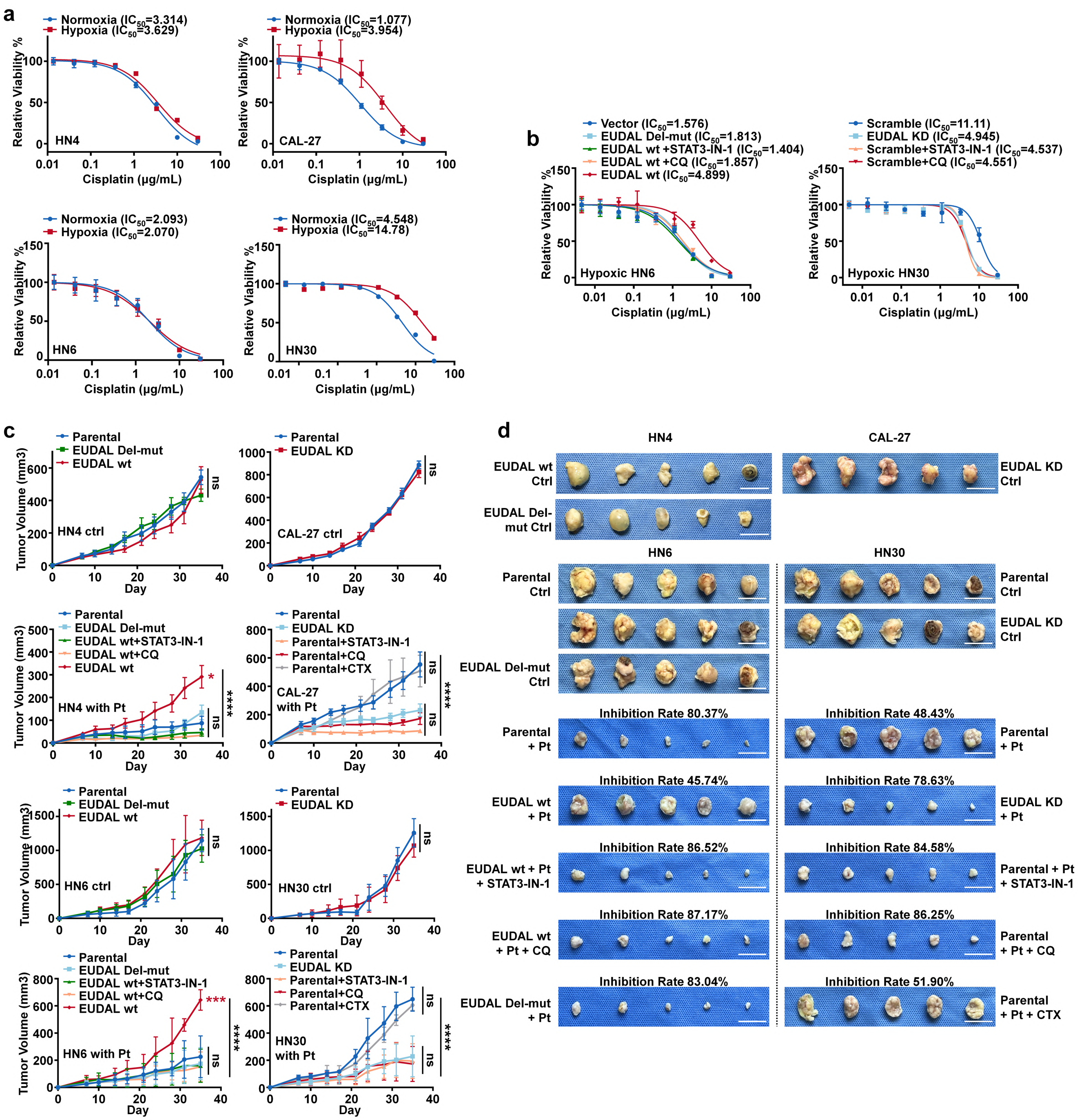
a
Normoxia (IC50=3.314)
Hypoxia (IC50=3.629)
HN4
Normoxia (IC50=1.077)
Hypoxia (IC50=3.954)
CAL-27
Normoxia (IC50=2.093)
Hypoxia (IC50=2.070)
HN6
Normoxia (IC50=4.548)
Hypoxia (IC50=14.78)
HN30
Relative Viability %
Cisplatin (μg/mL)
b
Vector (IC50=1.576)
EUDAL Del-mut (IC50=1.813)
EUDAL wt +STAT3-IN-1 (IC50=1.404)
EUDAL wt +CQ (IC50=1.857)
EUDAL wt (IC50=4.899)
Hypoxic HN6
Scramble (IC50=11.11)
EUDAL KD (IC50=4.945)
Scramble+STAT3-IN-1 (IC50=4.537)
Scramble+CQ (IC50=4.551)
Hypoxic HN30
c
Parental
EUDAL Del-mut
EUDAL wt
HN4 ctrl
EUDAL KD
CAL-27 ctrl
EUDAL wt+STAT3-IN-1
EUDAL wt+CQ
HN4 with Pt
Parental+STAT3-IN-1
Parental+CQ
Parental+CTX
CAL-27 with Pt
HN6 ctrl
HN30 ctrl
HN6 with Pt
HN30 with Pt
Tumor Volume (mm3)
Day
ns
*
***
****
d
HN4
CAL-27
EUDAL wt Ctrl
EUDAL KD Ctrl
EUDAL Del-mut Ctrl
HN6
HN30
Parental Ctrl
EUDAL KD Ctrl
EUDAL Del-mut Ctrl
Inhibition Rate 80.37%
Inhibition Rate 48.43%
Parental + Pt
Inhibition Rate 45.74%
Inhibition Rate 78.63%
EUDAL wt + Pt
EUDAL KD + Pt
Inhibition Rate 86.52%
Inhibition Rate 84.58%
EUDAL wt + Pt + STAT3-IN-1
Parental + Pt + STAT3-IN-1
Inhibition Rate 87.17%
Inhibition Rate 86.25%
EUDAL wt + Pt + CQ
Parental + Pt + CQ
Inhibition Rate 83.04%
Inhibition Rate 51.90%
EUDAL Del-mut + Pt
Parental + Pt + CTX

Fig. S11 Hypoxia confers tumor cells drug resistance through STAT3-mediated induction of autophagy. **a** Hypoxia treatment induced resistance of CAL-27 and HN30 to cisplatin but had no effect on drug resistance in HN4 and HN6. **b** IC_{50} values for cisplatin were determined in hypoxic HN6 and HN30 receiving indicated treatments. **c-d** Tumor-bearing mice models were established by inoculating indicated cell lines. Xenografts were treated with cisplatin alone or in combination with STAT3-IN-1, CQ, or cetuximab. Tumor volumes (**c**) and tumor inhibition rates (**d**) were determined in each experimental group. $n = 5$ for IC_{50} estimation, $n = 5$ for *in vivo* animal experiments. Data are represented as mean ± SD. ns, no significance; *, $p < 0.05$; **, $p < 0.01$; ***, $p < 0.001$; ****, $p < 0.0001$; unpaired Student's t-test or one-way ANOVA with Turkey's Honestly Significant Difference test (**c**). CTX, cetuximab; Pt, cisplatin; wt, wild-type; EUDAL Del-mut, a lncRNA mutant with an EGFR-binding motif deletion (Δ254-305 nt); KD, knockdown.

Figure S12:

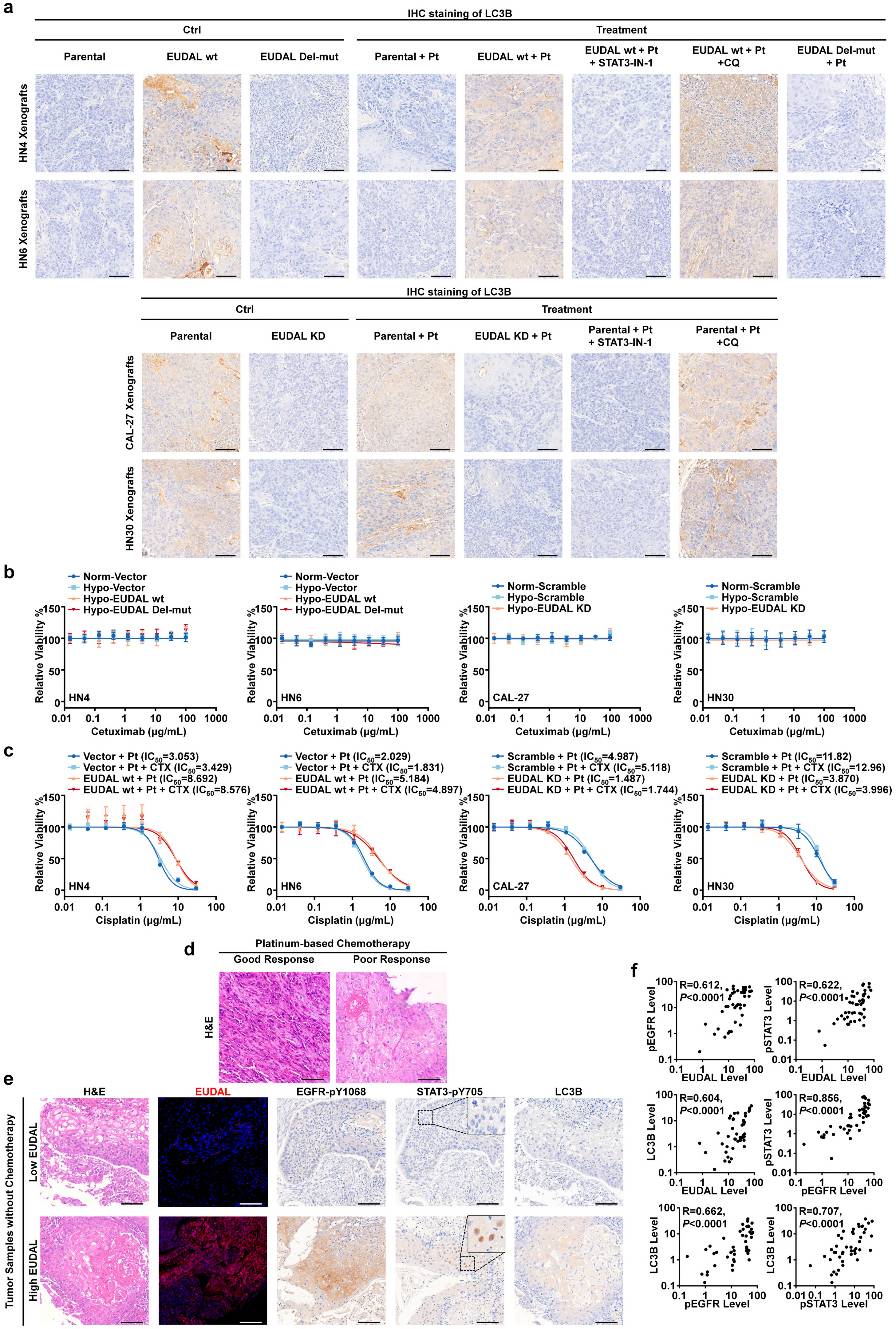

a
IHC staining of LC3B
Ctrl
Treatment
Parental
EUDAL wt
EUDAL Del-mut
Parental + Pt
EUDAL wt + Pt
EUDAL wt + Pt + STAT3-IN-1
EUDAL wt + Pt +CQ
EUDAL Del-mut + Pt
HN4 Xenografts
HN6 Xenografts
IHC staining of LC3B
Ctrl
Treatment
Parental
EUDAL KD
Parental + Pt
EUDAL KD + Pt
Parental + Pt + STAT3-IN-1
Parental + Pt +CQ
CAL-27 Xenografts
HN30 Xenografts
b
Norm-Vector
Hypo-Vector
Hypo-EUDAL wt
Hypo-EUDAL Del-mut
Norm-Scramble
Hypo-Scramble
Hypo-EUDAL KD
Relative Viability %
HN4
HN6
CAL-27
HN30
Cetuximab (μg/mL)
c
Vector + Pt (IC50=3.053)
Vector + Pt + CTX (IC50=3.429)
EUDAL wt + Pt (IC50=8.692)
EUDAL wt + Pt + CTX (IC50=8.576)
Vector + Pt (IC50=2.029)
Vector + Pt + CTX (IC50=1.831)
EUDAL wt + Pt (IC50=5.184)
EUDAL wt + Pt + CTX (IC50=4.897)
Scramble + Pt (IC50=4.987)
Scramble + Pt + CTX (IC50=5.118)
EUDAL KD + Pt (IC50=1.487)
EUDAL KD + Pt + CTX (IC50=1.744)
Scramble + Pt (IC50=11.82)
Scramble + Pt + CTX (IC50=12.96)
EUDAL KD + Pt (IC50=3.870)
EUDAL KD + Pt + CTX (IC50=3.996)
Relative Viability %
Cisplatin (μg/mL)
d
Platinum-based Chemotherapy
Good Response
Poor Response
H&E
e
Tumor Samples without Chemotherapy
Low EUDAL
High EUDAL
H&E
EUDAL
EGFR-pY1068
STAT3-pY705
LC3B
f
R=0.612, P<0.0001
R=0.622, P<0.0001
R=0.604, P<0.0001
R=0.856, P<0.0001
R=0.662, P<0.0001
R=0.707, P<0.0001
pEGFR Level
pSTAT3 Level
LC3B Level
EUDAL Level

Fig. S12 LC3B levels in tumor xenografts, therapeutic effects of cetuximab *in vitro* and *in vivo*, and EUDAL/EGFR/STAT3/autophagy signal axis in untreated primary tumors. **a** Representative immunohistochemistry staining for LC3B in tumor xenografts of indicated treatments. **b** IC_{50} of cetuximab alone in HN4, HN6, HN30, and CAL-27 cells after indicated treatments. **c** IC_{50} of cisplatin treatment with or without cetuximab combination in HN4, HN6, HN30, and CAL-27 cells after indicated treatments. **d** Representative hematoxylin and eosin (H&E) staining of slides in Fig. 10a. **e** Representative hematoxylin and eosin (H&E) staining, immunohistochemistry staining for pEGFR, pSTAT3, and LC3B, and RNA FISH staining for EUDAL in tumor tissues from patients without chemotherapy. **f** Correlations among EUDAL, pEGFR, pSTAT3, and LC3B levels in tumor tissues. Data were from representative results of at least three independent experiments. Data are represented as mean ± SD. Spearman rank correlation (**f**). Pt, cisplatin; CTX, cetuximab; wt, wild-type; EUDAL Del-mut, a lncRNA mutant with an EGFR-binding motif deletion (Δ254-305 nt); KD, knockdown.

SUPPLEMENTARY TABLES

Supplementary Table S1: Primers used for PCR

Target	Primer	Sequence
EGFR exon 18	Forward	AGGCGTGGAAACAGACATAG
	Reverse	TTGGTCTCACAGGACCACTG
EGFR exon 19	Forward	CCCCAGCAATATCAGCCTTAG
	Reverse	GCCAGTAATTGCCTGTTTCC
EGFR exon 20	Forward	TCTCCCACTGCATCTGTCAC
	Reverse	TTGATGAGAGTTTCCACATGC
EGFR exon 21	Forward	TCTTTCATGCGCCTTTCC
	Reverse	TGCAGGGAGAGACTGAAACC

Supplementary Table S2: Densitometric analysis Results

Group	Densitometric measurements				
**Fig. S2c**					
CHX Time (h)	0	3	6	12	24
HN4 CHX	1	0.77636	0.69448	0.57889	0.33415
CAL-27 CHX	1	0.99105	0.90268	0.79706	0.86706
HN6 CHX	1	0.59849	0.59334	0.45393	0.31242
HN30 CHX	1	0.91948	0.93232	0.90292	0.90616
**Fig. S2e-S2f**					
CHX Time (h)	0	3	6	12	24
HN4 CHX	1	0.53651	0.34336	0.30981	0.30641
HN4 CHX+Baf	1	1.10634	0.94864	1.07757	1.10080
HN4 CHX+MG132	1	0.59801	0.60962	0.36537	0.31354
HN6 CHX	1	0.53353	0.37725	0.32134	0.30817
HN6 CHX+Baf	1	0.95314	0.90885	0.99972	1.14043
HN6 CHX+MG132	1	0.39991	0.42198	0.29949	0.28096
**Fig. S5a**					
CHX Time (h)	0	3	6	12	24
HN4 Vector	1	0.68372	0.54565	0.52106	0.35282
HN4 EUDAL wt	1	0.98782	0.93152	1.00031	0.92418
HN4 EUDAL Del-mut	1	0.58432	0.53486	0.59781	0.34109
HN6 Vector	1	0.77170	0.49285	0.40509	0.26092
HN6 EUDAL wt	1	1.00074	0.96645	0.92945	0.88816
HN6 EUDAL Del-mut	1	0.85357	0.64141	0.31892	0.34921
CAL-27 Scramble	1	0.97235	0.93161	0.90105	1.00326
CAL-27 EUDAL KD	1	0.58098	0.46842	0.24057	0.25801
HN30 Scramble	1	0.97552	0.95692	0.89590	0.86694
HN30 EUDAL KD	1	0.81246	0.56737	0.39893	0.23950

**Fig. S7b**					
Hypoxia Time (h)	0	6	12	24	48
HN4 pY1068	1	1.03431	1.17636	1.31059	1.36526
CAL-27 pY1068	1	1.71086	2.19805	2.19299	3.97406
HN6 pY1068	1	1.12030	1.10780	1.00980	1.06883
HN30 pY1068	1	1.35350	2.06087	3.50827	3.48210
HN4 pSTAT3	1	0.91957	0.79103	0.93782	0.91370
CAL-27 pSTAT3	1	1.03106	1.25644	1.50314	2.44309
HN6 pSTAT3	1	0.86427	0.93420	0.82063	0.79514
HN30 pSTAT3	1	0.70467	1.51029	1.93139	2.75258
HN4 pSTAT1	1	1.18385	1.15493	0.94880	0.79712
CAL-27 pSTAT1	1	1.05194	1.14886	1.12618	0.94252
HN6 pSTAT1	1	1.05624	0.94116	1.08619	1.33923
HN30 pSTAT1	1	0.82067	0.92304	1.22411	1.01104
HN4 pERK	1	0.73507	0.61951	0.65642	0.52746
CAL-27 pERK	1	0.49537	0.52863	0.29633	0.21504
HN6 pERK	1	1.00141	0.91956	0.67834	0.78109
HN30 pERK	1	1.00654	1.09385	0.81123	0.83449
HN4 pAKT	1	0.38930	0.63081	0.70185	0.65249
CAL-27 pAKT	1	0.52953	0.62176	0.54356	0.13767
HN6 pAKT	1	0.86734	0.78532	0.45996	0.63786
HN30 pAKT	1	1.09316	1.02761	1.14001	0.98703
**Fig. S7c-S7d**					
Hypoxia Time (h)	0	6	12	24	48
HN4 Vector	1	0.74263	0.56300	0.48909	0.50652
HN4 EUDAL wt	1	1.05419	1.33456	2.19906	2.38547
HN4 EUDAL Del-mut	1	1.35751	1.09805	0.66051	0.56510
CAL-27 Scramble	1	2.62837	3.48602	3.94986	3.58146

CAL-27 EUDAL KD	1	0.79232	0.89924	1.18928	1.28151
HN6 Vector	1	0.91350	0.80954	0.82627	1.08838
HN6 EUDAL wt	1	1.79953	2.28806	2.81784	3.34384
HN6 EUDAL Del-mut	1	0.66299	0.82982	1.17463	1.31572
HN30 Scramble	1	1.63719	2.20744	2.31233	3.17403
HN30 EUDAL KD	1	0.91185	0.72624	0.74293	0.72185

Supplementary Table S3: Primers used for q-PCR

Target	Primer	Sequence
ACTB	Forward	CATGTACGTTGCTATCCAGGC
	Reverse	CTCCTTAATGTCACGCACGAT
ENST00000414640	Forward	GCATCATTGGTCTGCCCTGT
	Reverse	CCTGGAGCGGTTACTTATTTTG
ENST00000517420	Forward	GAAAGGACCCAACAGGGAGTG
	Reverse	TCTTAGGTTCGGATGTAGGAGAAG
ENST00000526951	Forward	CCGCCCTAGATATACTCAGATCC
	Reverse	CCTCCTCTCCCTTCCTACACTC
ENST00000569274	Forward	GATACCAAGTGATTCCAAACACAC
	Reverse	GGCTAGTCATAGATGGCATACCTAC
ENST00000585496	Forward	GCTAGACGCACGGACGATCA
	Reverse	GCATAAAAGGCTTCGCGG
ENST00000577847	Forward	AGACTACAGAGATACCAGGGTTGTG
	Reverse	CAAGGTGTCAGCAGGTTTGG
uc003nog.1	Forward	GGCTGAGGGCAAGTGAATAAATC
	Reverse	CACACCACCTTTGAGGAGTTAAGC
ChIP-NC	Forward	GGTTCTTTCCACGCCCTTCAGG
	Reverse	GGAGCATTGTAGAGCAGTAGCCATG
ChIP-HRE1	Forward	CACAGCCTTCCCCAGCATTCAC
	Reverse	CCTGGGAGGAAGAGGACAGACTG
ChIP-HRE2	Forward	GCCTGCATTTGGCTTGTCCTATTTG
	Reverse	TCCCAAAGAAGACCCAGGCTGAG
ChIP-HRE3	Forward	CCCTTGTGGGGCTGTTCAAA
	Reverse	TTCGGTACCTTTTAAATTCCTGCAT
ChIP-HRE4	Forward	TGGCTCCAGGCAGGTACACAG
	Reverse	GGGGAAGCTGGTGAAGGAAGTTG

Supplementary Table S4: Clinicopathological information of included patients

	**Good Response (n = 22)**	**Poor Response (n = 23)**	***p* value†**
**Systemic Therapy Response**			
	CR (n = 1)	SD (n = 14)	
	PR (n = 21)	PD (n = 9)	
**Age (years)**			**0.783‡**
Mean (SD)	58.3 (10.4)	57.5 (8.8)	
**Gender**			**0.121**
Male	18	14	
Female	4	9	
**Presentation**			**0.339**
Primary	18	16	
Recurrent	4	7	
**Pathological Grade**			**0.377**
I	4	2	
II	13	18	
III	5	3	
**Tumor Size (cm)**			**0.105‡**
Mean (SD)	3.76 (1.43)	3.04 (1.51)	
**Lymphnode Metastasis**			**0.666**
Positive	16	18	
Negative	6	5	
**Extranodal Extension**			**0.793**
Positive	5	6	
Negative	17	17	
**Perinural Invasion**			**0.396**
Positive	5	3	
Negative	17	20	

† Pearson Chi-square test; ‡ Unpaired Student's *t*-test.

Supplementary Table S5: RNA interference target sequences

Target	Sequence
RAB11	GCCTTATTGGTTTATGACATT
EGFR	CATCAGTGGCGATCTCCACAT
HIF-1α	CUAACUGGACACAGUGUGUTT
BNIP3	GGAAAGAAGTTGAAGCA
EUDAL	GCAAAGGATTCGTCAGAAA
